# Computational Studies of Coinage Metal Anion M^−^ + CH_3_X (X = F, Cl, Br, I) Reactions in Gas Phase

**DOI:** 10.3390/molecules27010307

**Published:** 2022-01-04

**Authors:** Fan Wang, Xiaoyan Ji, Fei Ying, Jiatao Zhang, Chongyang Zhao, Jing Xie

**Affiliations:** 1 Key Laboratory of Cluster Science of Ministry of Education, School of Chemistry and Chemical Engineering, Beijing Institute of Technology, Beijing 100081, China; bitwangfan@bit.edu.cn (F.W.); xiaoyanji@bit.edu.cn (X.J.); yingfei2020@bit.edu.cn (F.Y.); zhangjt@bit.edu.cn (J.Z.); 2 Beijing Key Laboratory of Construction-Tailorable Advanced Functional Materials and Green Application, School of Materials Science & Engineering, Beijing Institute of Technology, Beijing 100081, China

**Keywords:** C−X bond activation, oxidative insertion, nucleophilic substitution reaction, natural bond orbital, halogen-bonded complex

## Abstract

We characterized the stationary points along the nucleophilic substitution (S_N_2), oxidative insertion (OI), halogen abstraction (XA), and proton transfer (PT) product channels of M^−^ + CH_3_X (M = Cu, Ag, Au; X = F, Cl, Br, I) reactions using the CCSD(T)/aug-cc-pVTZ level of theory. In general, the reaction energies follow the order of PT > XA > S_N_2 > OI. The OI channel that results in oxidative insertion complex [CH_3_–M–X]^−^ is most exothermic, and can be formed through a front-side attack of M on the C-X bond via a high transition state OxTS or through a S_N_2-mediated halogen rearrangement path via a much lower transition state invTS. The order of OxTS > invTS is inverted when changing M^−^ to Pd, a d^10^ metal, because the symmetry of their HOMO orbital is different. The back-side attack S_N_2 pathway proceeds via typical Walden-inversion transition state that connects to pre- and post-reaction complexes. For X = Cl/Br/I, the invS_N_2-TS’s are, in general, submerged. The shape of this M^−^ + CH_3_X S_N_2 PES is flatter as compared to that of a main-group base like F^−^ + CH_3_X, whose PES has a double-well shape. When X = Br/I, a linear halogen-bonded complex [CH_3_−X∙··M]^−^ can be formed as an intermediate upon the front-side attachment of M on the halogen atom X, and it either dissociates to CH_3_ + MX^−^ through halogen abstraction or bends the C-X-M angle to continue the back-side S_N_2 path. Natural bond orbital analysis shows a polar covalent M−X bond is formed within oxidative insertion complex [CH_3_–M–X]^−^, whereas a noncovalent M–X halogen-bond interaction exists for the [CH_3_–X∙··M]^−^ complex. This work explores competing channels of the M^−^ + CH_3_X reaction in the gas phase and the potential energy surface is useful in understanding the dynamic behavior of the title and analogous reactions.

## 1. Introduction

The activation of C−X bonds has an essential role in many catalytic industrial processes involving organic substrates, generating value-added compounds [1,2,3]. Extensive experimental and theoretical research has been conducted, mainly focusing on the oxidative addition of C−X bond by the meal [4,5,6]. A variety of mechanisms were proposed for the oxidative addition. Crespo et al. categorized the mechanisms into the ionic, bimolecular nucleophilic substitution (S_N_2), concerted, and atom transfer types [7]. Efforts were put to understanding the mechanisms and search for suitable catalysts for C−X bond activation [8,9,10,11,12,13]. The discovery of catalytic gold by Haruta et al. stimulated the development of gold catalysts in the form of surfaces, nanoparticles, embedded clusters, and single atoms [14,15,16,17,18]. The nucleophilicity of gold is important in oxidative reactions, the Au^−^ anion is more nucleophilic than Au(0), Au(I), and Au(III) species, and thus reactions of atomic Au^−^ anions with hydrocarbons in the gas phase were studied [14,19]. The study of gas-phase ion-molecule reactions provides mechanistic insights into the bond-breaking and -making steps that are often obscured under condensed-phase environments, which are complicated by solvents, counterions, and ligands [14,20,21,22,23,24,25,26,27,28,29]. Although gas-phase obtained information cannot replace that of the condensed phase, understanding the intrinsic unit makes it more feasible to improve chemical reactions or catalysts from a bottom-up approach [20,22,26,30,31].

In the gas-phase reaction of Au^−^ + CH_3_I, Wilkins’ group observed the formation of I^−^ via a nucleophilic substitution (S_N_2) reaction [14]. Tsukuda’s group recently isolated an AuCH_3_I^−^ adduct by collision cooling under high helium pressure, and combined photoelectron spectroscopy and computational studies confirmed the adduct is an oxidative insertion complex [CH_3_−Au−I]^−^, which is formed via an S_N_2-mediated mechanism [19]. Later, Tsukuda’s group extended their studies to Ag and Cu, and the Grignard reagent-like [CH_3_−M−I]^−^ complex was also found [30]. It is known that the dynamics of gas-phase reactions are complex for a system that may deviate from the stationary PES [31]. In previous studies of gas-phase Y^−^ + CH_3_X reactions, where Y refers to non-metallic anions such as halogen anions, HO^−^, HS^−^, NH_2_^−^, CN^−^, etc., in addition to multiple product channels, multiple dynamical mechanisms for the major S_N_2 product channel were discovered, including roundabout, double-inversion, hydrogen/halogen-bonded complex mediated mechanisms [24,25,31,32,33]. Hence, it is intriguing to explore the competing product channel and mechanisms for metallic nucleophile M^−^ reacting with CH_3_I reactions.

**Scheme 1:**
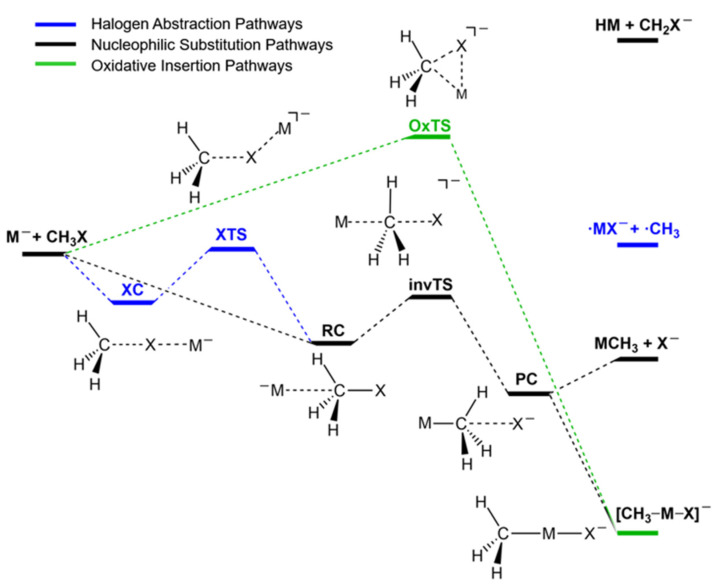
The potential energy profile scheme for the M^−^ + CH_3_X reaction in the gas phase that displays the proton transfer, halogen abstraction, nucleophilic substitution, and oxidative addition pathways.

In this study, a complicated PES is constructed for the M^−^ + CH_3_X reaction, where M = Cu, Ag, and Au. As shown in Scheme 1, four product channels including the nucleophilic substitution (S_N_2) channel, the oxidative insertion (OI) channel, the proton transfer (PT) channel, and halogen-abstraction (XA) channels were considered. Depending on the attack direction of M^−^, the front-side attack on C−I bond and on the I atom and the back-side attack on C atom mechanisms were addressed in detail. We also investigated the role of leaving the group by changing CH_3_I to CH_3_Br, CH_3_Cl, and CH_3_F. Comparisons were made between the PES properties of the current reaction and analogue reactions with the d^10^-metal atom Pd and main-group base nucleophiles F^−^ [25,34,35].

## 2. Computational Methods

The structures were optimized with hybrid functional M06-2X [36], with the aug-cc-pVTZ basis set for the H, C, F and Cl atoms and the aug-cc-pVTZ-PP basis set for the Br, I, Cu, Ag, and Au atoms [37]. Vibrational frequencies were computed to confirm the stationary point nature of the local minimum (no imaginary frequency) and the transition states (one imaginary frequency) on the potential energy surface. Intrinsic reaction coordinates (IRC) were performed on each transition state to confirm their minimum energy path. On top of the M06-2X optimized structures, single point energies were calculated using the CCSD(T) method [34,35] with the same basis set. We denote this combined method as the CCSD(T)// M06-2X/aug-cc-pVTZ(-PP) method. If not specified, the values reported in this work refer to CCSD(T) corrected enthalpies at 298.15 K, where additional energetic values are available in the Supplementary Materials. A few structures were optimized with functionals other than M06-2X due to the failure of convergence, and they are also specified in the Supplementary Materials [38,39]. Energetic values given by M06-2X/aug-cc-pVTZ(-PP) method are also provided in the Supplementary Materials, Tables S1 and S2, for interested readers. Atomic charges were evaluated by natural population analysis (NPA) (Table S3) based on the natural bond orbital (NBO) scheme [40,41]. T_1_ diagnostic [42] and CASSCF [43] calculations were applied to selected species and confirmed the single reference nature of the metal-involved species (Table S4, Figure S1). CASSCF calculation methods [43,44,45,46,47,48,49,50,51] and results are provided in the Supplementary Materials. All computations were performed using Gaussian 09 software [52].

## 3. Results and Discussion

### 3.1. Reaction Enthalpies

Table 1 lists the reaction enthalpies of four product channels of titled reactions. Take the Cu^−^ + CH_3_I reaction, for example, the reaction enthalpies are in a decreasing order of proton transfer (PT, 41.4 kcal/mol) > halogen abstraction (XA, −9.1 kcal/mol) > nucleophilic substitution (S_N_2, −37.6 kcal/mol) > oxidative insertion (OI, −82.1 kcal/mol). The proton transfer (PT) product channel is highly endothermic, implying that only high collision energy or photoexcitation can induce this channel. The halogen abstraction (XA) channel is slightly exothermic (−9.1, −7.2, −5.1 kcal/mol) when the substrate is CH_3_I, CH_3_Br, or CH_3_Cl, but when the substrate is CH_3_F this channel becomes endothermic by 12.3 kcal/mol. Similarly, the S_N_2 channel is exothermic for X = Cl, Br, and I, and becomes slightly endothermic for X = F (4.2 kcal/mol). As for the OI channel, it is highly exothermic for all the leaving groups, i.e., −64.3, −79.8, −81.2, −82.1 kcal/mol for X = F, Cl, Br, and I, respectively. The order of reaction enthalpies, being PT > XA > S_N_2 > OI, applies to the case of Ag^−^ and Au^−^ being the nucleophiles. When Ag^−^/Au^−^ anions are the nucleophiles, the reaction enthalpy values of each product channel are higher than in the case of Cu^−^.

### 3.2. Potential Energy Profiles

**Figure 1:**
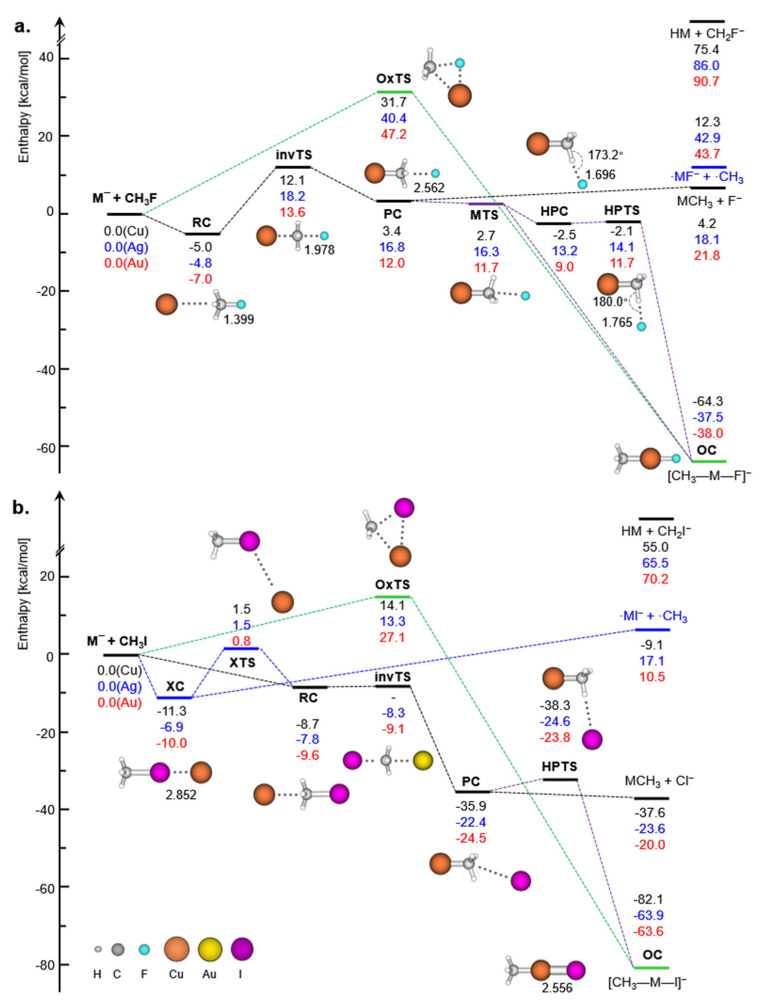
Potential energy profile of (**a**) a M^−^ + CH_3_F reaction, and (**b**) a M^−^ + CH_3_I reaction. Enthalpy values (in kcal/mol) at 298.15 K are reported. Selected bond distances (Å) and angles (°) are displayed for stationary points of the Cu^−^ + CH_3_F/CH_3_I system.

Figure 1 depicts the potential energy surfaces (PESs) for Cu^−^ + CH_3_F and Cu^−^ + CH_3_I reactions with back-side attack, front-side attack at the C−X bond and front-side attack at X atom mechanisms. Values for M = Ag/Au are also listed. Additional PESs for M^−^ + CH_3_Cl/CH_3_Br and selected structures are presented in the Supplementary Materials, Figures S2–S4, and the bond metrics are provided in Table S5. In the following, we will use Cu^−^ + CH_3_Cl and CH_3_I for illustration.

#### 3.2.1. The Back-Side Attack Pathway

The traditional back-side attack proceeds with M^−^ anion approaches C atom and forms a prereaction complex, M^−^∙∙∙CH_3_X (RC). Then, it crosses a Walden-inversion transition state [M∙·CH_3_∙·X]^−‡^ (invTS), which connects to a postreaction complex MCH_3_∙∙∙X^−^ (PC) before it dissociates to the S_N_2 products MCH_3_ and X^−^. All the RCs are ion-dipole complexes that adopt a linear M-C-X shape and are lower in energy than reactants. When the nucleophile is Cu^−^, the depth of the RC wells increases from 5.0 (X = F) to 6.5 (Cl), 7.2 (Br), and 8.7 (I) kcal/mol. For the transition state invTS, the barriers decrease from positive to submerged values, as leaving group X changes from F to I, with respective values of 12.1 (F), −3.1 (Cl), −6.8 (Br), and −9.3 (I) kcal/mol. The vibrational mode of the imaginary frequency of invTS involves the umbrella-inversion of the CH_3_-group, and the corresponding imaginary frequencies are i501, i413, i320, and i31 cm^−1^, respectively. We noted that the imaginary frequency of invTS of the Cu^−^ + CH_3_I reaction, being i31 cm^−1^, representsinga half umbrella-inversion of CH_3_-group, is too low, and attempts to search for the correct invTS were not successful using multiple DFT and basis sets. The corresponding imaginary frequency values for the cases of Ag^−^ and Au^−^ as nucleophiles are provided in Table S6.

Similar to the RCs, the PCs are also ion-dipole complexes with linear M-C-X shapes, and the only exceptions are CuCH_3_∙∙∙I^−^ and AgCH_3_∙∙∙I^−^, whose M-C-I angles are 160° and 166° (Table S5). As listed in Table 2, when M = Cu/Ag and X = Cl, Br, I, the enthalpies of PCs are slightly higher than those of S_N_2 products. For instance, CuCH_3_∙∙∙I^−^ (−35.9 kcal/mol) is 1.7 kcal/mol higher than products CuCH_3_ + I^−^ (−37.6 kcal/mol). In other cases, when X = F for all metals and the combination of M = Au and X = Cl, Br, I, the PCs are lower in energy than S_N_2 products, forming a shallow well before the products. For instance, AuCH_3_∙∙∙I^−^ (−24.5 kcal/mol) is 4.5 kcal/mol lower than the products of AuCH_3_ + I^−^ (−20.0 kcal/mol).

As for X = F, besides the traditional linear ion-dipole PC, a hydrogen-bonded postreaction complex (HPC) can be formed. As shown in Figure 1a, a hydrogen bond is formed between the H atom and F atom in the CuH_2_CH∙∙∙F^−^ (HPC) complex, with a ∠C-H-F angle of 173.2° and an H-F distance of 1.696 Å. Almost all the PCs and HPCs for X = F are higher in energy than the reactants. For the Cu^−^ + CH_3_F system, CuCH_3_∙∙∙F^−^ (PC, 3.4 kcal/mol) can convert to CuH_2_CH∙∙∙F^−^ (HPC, −2.5 kcal/mol) via the rotation of the F atom to cross transition state MTS (2.7 kcal/mol). Then, by continuing the rotating of F atom, the HPC will finally convert to an oxidative insertion complex (OC) product [CH_3_–Cu–F]^−^. A transition state that we denoted as HPTS can be located on the exit channel, and the barrier of 0.4 kcal/mol is almost neglectable in comparison to the sharp drop in energy to the OC complex [CH_3_–Cu–F]^−^ (−64.3 kcal/mol). Notably, the OC complex is formed after nucleophilic substitution via Walden-inversion, so the stereo geometry of the CH_3_-group is inverted, and we denote this mechanism as the S_N_2-mediated oxidative insertion mechanism.

Although we were unable to locate HPC for systems with M = Cu/Ag and X = Cl, Br, I, a small perturbation may drive the linear PC complex MCH_3_∙∙∙X^−^ from its equilibrium geometry. Provided additional collisions remove energies from the PC complex before it dissociates to the S_N_2 products MCH_3_ + X^−^, it will convert to the more stable oxidative insertion products, [CH_3_–M–X]^−^. In fact, during our optimization, the search for some PCs and HPCs produced the OCs [CH_3_–M–X]^−^.

#### 3.2.2. The Front-Side Attack at C Atom Pathway

In the second mechanism, the nucleophile M^−^ attacks the front-side of CH_3_X at the carbon atom by crossing a transition state denoted as OxTS, resulting in an oxidative insertion [CH_3_–M–X]^−^ product. Within the OxTS structure, the incoming nucleophile M^−^ and the leaving group X are on the same side of CH_3_-group, while the CH_3_-group has a nearly planar shape and the ∠M-C-X angle is less than 90°. Taking the Cu^−^ + CH_3_X system, for example, the respective ∠Cu-C-X angles are 51.4° (F), 75.6° (Cl), 78.0° (Br), and 82.8° (I). The vibrational mode of the imaginary frequency involves the CH_3_ shift from X to M, along with X-C bond elongation and M-C bond shrinking, and the imaginary frequencies were i538 (F), i429 (Cl), i371 (Br), i326 (I) cm^−1^, respectively. Unlike the S_N_2-mediated OI mechanism, this front-side attack (FSA) pathway retains the stereo geometry of CH_3_ group, as illustrated in the IRC calculation in Figure S5.

**Figure 2:**
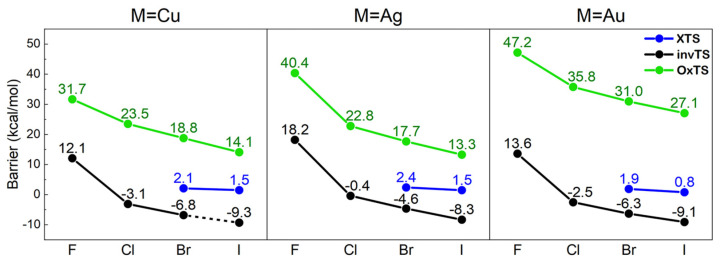
Barrier heights of the transition states invTS, OxTS, and XTS of M^−^ + CH_3_I reactions. Enthalpy values at 298.15 K are reported using the CCSD(T)//M06-2X/aug-cc-pVTZ(-PP) method.

**Figure 3:**
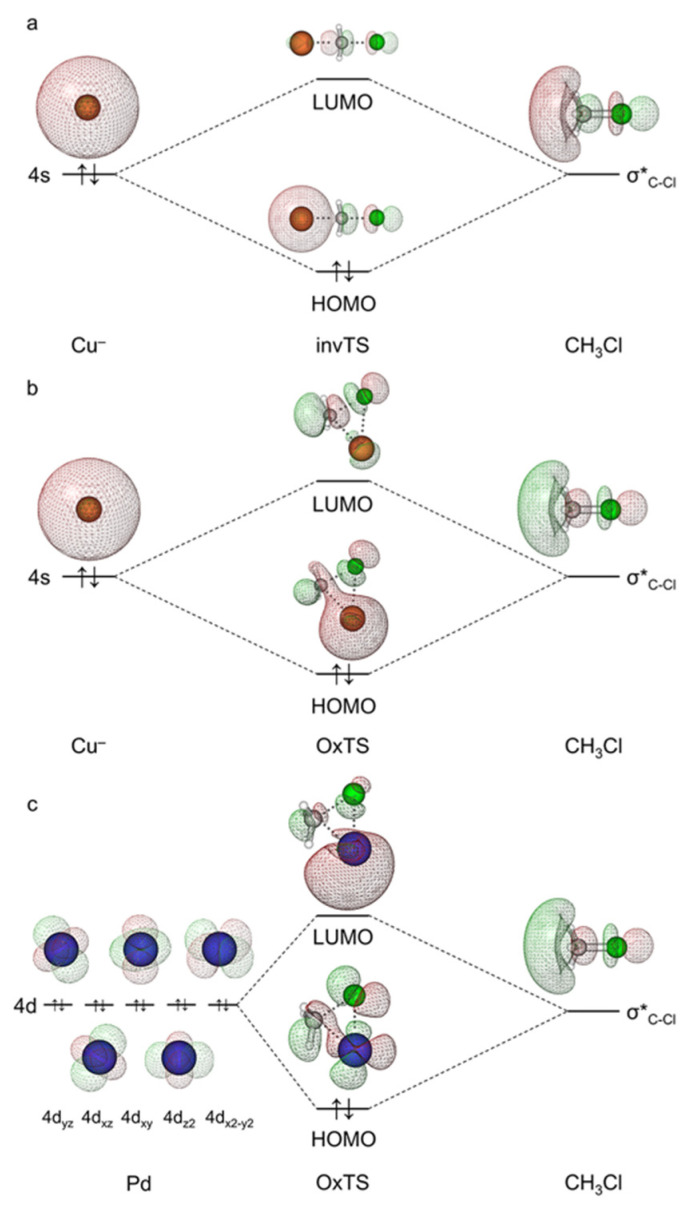
Molecular orbital schemes for the (**a**) back-side attack (invTS) and (**b**) front-side attack (OxTS) transition states of Cu^–^ + CH_3_Cl reactions, as compared to (**c**) the OxTS of Pd + CH_3_Cl reaction. Color code: H, white; C, gray; Cl, green; Pd, dark blue.

However, this FSA path has much higher barrier than the back-side attack path. For the OxTS transition state of the Cu^−^ + CH_3_X system, the barrier heights were 31.7 (F), 23.5 (Cl), 18.8 (Br), and 14.1 (I) kcal/mol, respectively. Figure 2 depicts the comparison of barrier heights between back-side attack and front-side attack for all the metal anions under study. Bickelhaupt et al. (1995) [53] reported the oxidative insertion of the Pd + CH_3_Cl reaction as a front-side S_N_2 substitution, and the oxidative insertion transition state (OxTS) was much lower than the S_N_2/Cl-rearrangement transition state, TS(S_N_2/Cl-ra). Energetic comparison at the CCSD(T)//M06-2X/aug-cc-pVTZ(-PP) level of theory also predicts the same order (Table S7 for details). This order was inverted in our M^−^ + CH_3_X case. This was because in the Pd + CH_3_Cl system, the d-type HOMO of Pd was suitable for front-side interaction with the σ*_C-Cl_ orbital of the substrate CH_3_Cl. However, the HOMO of M^−^ (M = Cu, Ag, Au) is s-orbital, and the interaction between this s-type HOMO and σ*_C-X_ orbital is poor because the HOMO lobe approaches on a nodal surface of σ*_C-X_, i.e., a front-side attack. On the contrary, this s-type HOMO favors interactions with the backside of the σ*_C-X_ orbital of the substrate (Figure 3). This means that Cu^−^/Ag^−^/Au^−^ anions behave more like a main group base, such as F^−^, that has p-type HOMO, and the backside attack is favored over frontside attack.

#### 3.2.3. The Halogen-Bonded Complex Pathway

Alternatively, the incoming nucleophile can attack the leaving group X from the front-side. When X = Br and I, a halogen-bonded complex [CH_3_–X∙∙∙M]^−^ can be formed with a linear C-X-M bond, it is known that when halogen atom, especially I and Br, binds to an electron withdrawing group, such as CF_3_ or CH_3_ in our case, a *σ*-hole that carries positive charge appears on the other side of halogen atom along the axis of the C–X bond. Although theory predicted the existence of the *σ*-hole in 2007 by Politzer’s group [54] and strategies were developed to stabilize anions with halogen bonding [55], the *σ*-hole has been observed by experiments in 4BrPhM molecules only recently [56]. As a result, the *σ*-hole attracts negatively charged species to form a halogen bond. Attempts to locate the halogen-bonded complex for X = Cl and F were unsuccessful. Because the radius of the Cl atom and the F atom are much smaller than Br and I, they are difficult to polarize, so they are less likely to form a halogen-bonded complex.

**Figure 4:**
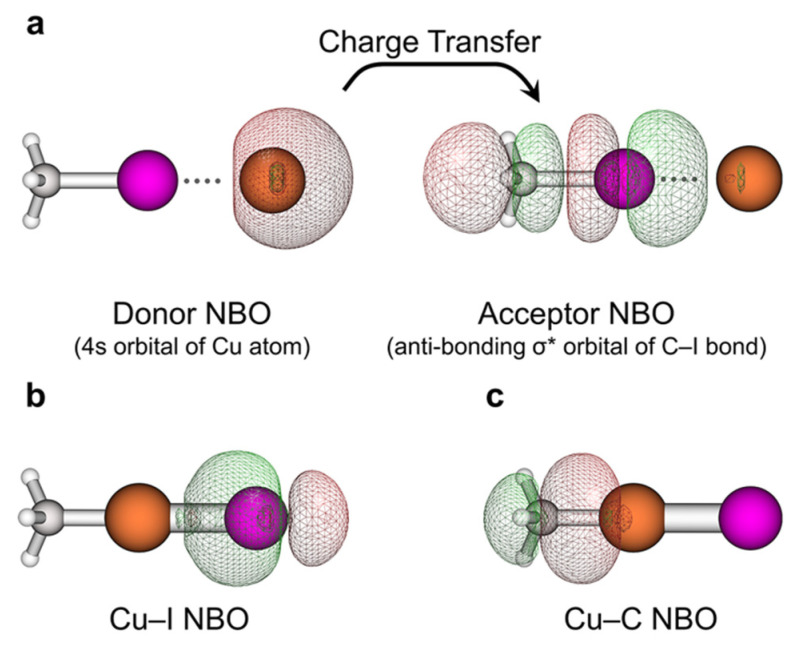
(**a**) Donor and acceptor natural bond orbitals (NBOs) of the halogen-bonded complex [CH_3_–I∙∙∙Cu]^−^ to illustrate the charge transfer interaction between I and Cu. NBOs of (**b**) Cu-I and (**c**) Cu-C in the oxidative addition complex [CH_3_–Cu–I]^−^.

To characterize the interaction between CH_3_X and M within the halogen-bonded complexes [CH_3_−X∙∙∙M]^−^, we performed natural bond orbital (NBO) calculations and analyzed the donor-acceptor charge transfer properties. Figure 4a depicts the interaction for [CH_3_−I∙∙∙Cu]^−^ as an example. The donor orbital is the Cu 4s orbital and the acceptor orbital is the σ* anti-bonding orbital of the C–I bond. Both orbitals align in the C–X bond direction and the donor-acceptor interaction is a *σ*-type interaction. Due to the charge transfer, the σ* anti-bonding orbital of the C–I bond becomes partially occupied, resulting in an elongation of the C-I bond in [CH_3_−I∙∙∙Cu]^−^ (2.350 Å) as compared with CH_3_I (2.140 Å). The halogen-bond interaction energy, which is evaluated as charge transfer stabilization energy under the NBO scheme (Table S8), was 56.6 kcal/mol for [CH_3_−I∙∙∙Cu]^−^.

Relative to the reactants, the enthalpies of the halogen-bonded complexes [CH_3_–I∙∙∙M]^−^ (XC) were −11.3 (M = Cu), −6.9 (Ag), and −10.0 kcal/mol (Au), respectively. The stability of the XCs were comparable to the prereaction complex RCs, but were much weaker than the oxidative insertion complexes [CH_3_–M–I]^−^ (OCs). For comparison, we also plotted the NBOs of the OCs. As shown in Figure 4b, an I-Cu bonding orbital is available within [CH_3_–Cu–I]^−^, showing a highly polarized nature towards the I atom. Orbital composition analysis shows that the I atom contributes ~90% and Cu contributes the remaining 10% to the I-Cu bond. For other X–M bonds in the OCs, the contributions of Cl, Br, I were almost the same, ~90%, and the contributions of F were higher, ~95%. See Table S9 for details.

Starting from the halogen-bonded complex [CH_3_–X∙∙∙M]^−^, the system can either break the C–X bond to form CH_3_ + XM^−^, or bend the C-X-M angle to proceed to the S_N_2-path. The halogen abstraction product channel is highly endothermic (as discussed in Section 3.1), and the latter path is more feasible. The transition states (XTS) that feature the bending of the C-X-M angle are found to be only slightly higher in energy than those of the reactants. For X = I, the enthalpies of XTSs are 1.5 (M = Cu), 1.5 (Ag), 0.8 kcal/mol (Au), respectively. However, the corresponding imaginary frequencies, i.e., i65 cm^−1^ (Cu), i63 cm^−1^ (Ag), and i66 cm^−1^ (Au), are very small, implying that exciting this mode is difficult. The system may dissociate to reactants instead of crossing the XTS. The IRC calculation shows that the XTS connects to the RC on the other side so that it will continue the S_N_2-path.

Overall, as shown in Figure 2, the order of barrier heights for the three pathways was OxTS > XTS > invTS for all three M^−^ and the four substrates when XTS is available. For each nucleophile M^−^, varying the leaving group from F to I decreases the barrier heights of all three transition states, consistent with the decreasing order of electronegativity of F > Cl > Br > I. The barrier heights of XTS and invTS were similar for M = Cu, Ag, and Au, but the values of OxTS were higher for Au^−^ than for Cu^−^ and Ag^−^. For instance, the barrier heights of OxTS for Cu^−^ + CH_3_X were 31.7 (X = F), 23.5 (Cl), 18.8 (Br), and 14.1 (I) kcal/mol, respectively, and the corresponding values for Au^−^ + CH_3_X were 47.2 (X = F), 35.8 (Cl), 31.0 (Br), and 27.1 (I) kcal/mol. Consequently, the barrier difference between OxTS and invTS enlarged greatly from ~23.8 kcal/mol for M = Cu to ~36.4 kcal/mol for M=Au.

#### 3.2.4. Comparison between Metallic Nucleophiles M^−^ and Main Group Nucleophiles F^−^

There appears to be a similarity between the M^−^ and main group base B as a nucleophile toward a reaction with CH_3_X substrate, as discussed in Section 3.2.2. Below, we use F^−^ as an example of a main group base, to compare with the PES of M^−^ reacting with CH_3_Cl.

**Figure 5:**
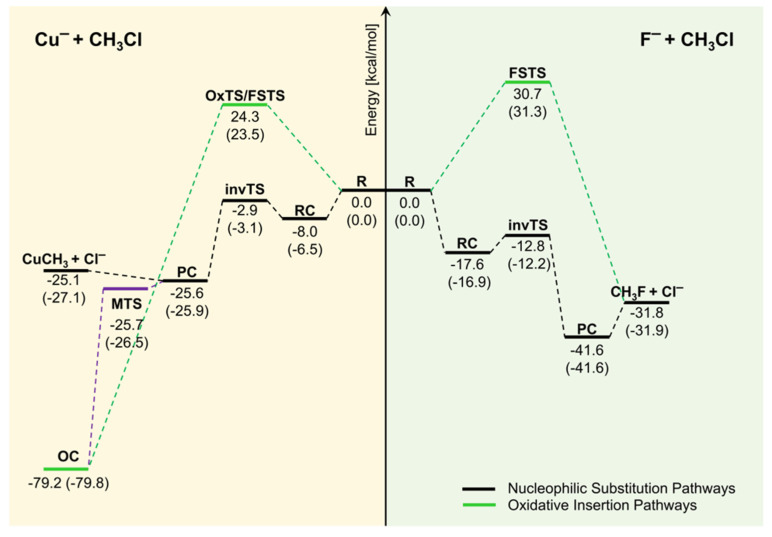
Potential energy profiles of (left) Cu^−^ + CH_3_Cl and (right) F^−^ + CH_3_Cl reactions. Energy values without ZPE are in normal text and enthalpy values at 298.15 K are in parentheses. The CCSD(T)//M06-2X/aug-cc-pVTZ(-PP) method was used for Cu^–^ + CH_3_Cl and the AE-CCSD(T)/aug-cc-pCVQZ method from [25] was used for F^–^ + CH_3_Cl.

As shown in Figure 5, the S_N_2 product channels of both F^−^ + CH_3_Cl and Cu^−^ + CH_3_Cl have comparable exothermicity, and the submerged invTS connects RC and PC. Clearly, the PES of F^−^ + CH_3_Cl has a double-well shape, but for the Cu^−^ + CH_3_Cl reaction the PC-well is flattened. A change of the substrate to CH_3_Br and CH_3_I further flattens the RC-well. This is one distinct feature among their S_N_2 PESs. As for the front-side attack transition state, IRC calculations show it connected to the oxidative insertion product [CH_3_–M–Cl]^−^ for Cu^−^ reactions with CH_3_Cl, but it connected to the S_N_2 products CH_3_F + Cl^−^ for the F^−^ reactions with CH_3_Cl. The outer p and d orbitals were available for M^−^ anions, making the formation of an oxidative insertion product [CH_3_–M–Cl]^−^ easy and favorable. However, this was not the case for the main group base B, which, like F, obeys the octet rule and is singly-valent. Dynamically, the OxTS of the Cu^−^ + CH_3_Cl system may end up with an S_N_2 product at high collision energy, when CuCH_3_ and Cl^−^ separate fast enough before forming Cu–Cl bond. It is anticipated that an ab initio molecular dynamics simulation would give more information on the dynamics of the reactions.

## 4. Conclusions

A comprehensive potential energy surface study has been completed for reactions of M^–^ + CH_3_X in the gas phase, where M refers to the coinage metals Cu, Ag, Au, and X = F, Cl, Br, and I. Four product channels including oxidative insertion (OI), nucleophilic substitution (S_N_2), halogen abstraction (XA), and proton transfer (PT) were considered. Both the front-side and back-side attack mechanisms were investigated. The key conclusions are listed below.

(1) In general, the reaction enthalpies of the four product channels are in a decreasing order of PT > XA > S_N_2 > OI, given that the oxidative insertion products [CH_3_–M–X]^–^ are thermodynamically most stable, and that the S_N_2 products CH_3_M + X^–^ the next-most stable.

(2) The oxidative insertion products [CH_3_–M–X]^–^ (OC) can be formed via two pathways. One is the front-side attack of M^–^ at the C-X bond that direct leads to OC and has a high barrier (OxTS). The other is an S_N_2-mediated halogen rearrangement pathway, which has a much lower barrier that crosses the typical back-side attack transition state (invTS). The latter path is kinetically favored. The order of OxTS > invTS is inverted when M^–^ is changed to a d-metal atom like Pd. Molecular orbital analysis illustrates the difference is raised from the differential symmetry of the attacking HOMO orbital, which is s-type for M^–^ and d-type for Pd.

(3) The front-side attack of M^–^ on the halogen atom of CH_3_I/CH_3_Br gives rise to a halogen-bonded complex [CH_3_–X∙∙∙M]^–^, which can bend the C-X-M bond to proceed along the S_N_2 path. Although there is M–X interaction within both the [CH_3_–M–X]^–^ (OC) and [CH_3_–X∙∙∙M]^–^ (XC) complexes, NBO analysis shows that the former represents a covalent bond, whereas the latter is a noncovalent interaction.

(4) Varying the leaving group from F to I decreases the reaction energy and barrier, consistent with the order of electronegativity of F > Cl > Br > I. No uniform trend is observed for when M changes from Cu to Ag and Au.

(5) Although the M^–^ nucleophile resembles a main-group base, like F^–^, as it reacts with CH_3_X, some differences were observed. The typical Walden-inversion PES is much flatter when the nucleophile is M^–^ than when it is F^–^. The most stable oxidative insertion complex [CH_3_–M–X]^–^ can only be formed for M^–^ thanks to the available outer orbitals.

This current study focuses on the stationary points on the PES of M^–^ + CH_3_X reaction. It is known that the reaction dynamics may deviate from the stationary PES and result in numerous dynamic mechanisms. It would be interesting to compare current studies with further dynamics simulations to expand our understanding of the atomistic mechanisms of titled reactions.

## Figures and Tables

**Table 1:** Calculated reaction enthalpies at 298.15 K of M^−^ + CH_3_X (M = Cu, Ag, Au; Y = F, Cl, Br, I) reactions. Values (in kcal/mol) are calculated with the CCSD(T)//M06-2X/aug-cc-pVTZ(-PP) method.

Products	X	F	Cl	Br	I
**M = Cu**					
OI	[CH_3_–Cu–X]^−^	−64.3	−79.8	−81.2	−82.1
S_N_2	CuCH_3_ + X^−^	4.2	−27.1	−31.9	−37.6
XA	CuX^−^ + CH_3_	12.3	−5.1	−7.2	−9.1
PT	HCu + CH_2_X^−^	62.5	49.8	46.3	41.4
**M = Ag**					
OI	[CH_3_–Ag–X]^−^	−37.5	−57.8	−61.0	−63.9
S_N_2	AgCH_3_ + X^−^	18.1	−13.1	−18.0	−23.6
XA	AgX^−^ + CH_3_	26.4	4.3	0.6	−3.0
PT	HAg + CH_2_X^−^	73.1	60.4	56.8	52.0
**M = Au**					
OI	[CH_3_−Au−X]^−^	−38.0	−57.3	−60.4	−63.6
S_N_2	AuCH_3_ + X^−^	21.8	−9.5	−14.3	−20.0
XA	AuX^−^ + CH_3_	43.7	20.7	15.9	10.5
PT	HAu + CH_2_X^−^	77.8	65.1	61.5	56.6

**Table 2:** Calculated electronic energies (E) and enthalpies at 298.15 K (H) of the stationary points on the potential energy profiles of M^−^ + CH_3_X reactions. Energy values (kcal/mol) are calculated with the CCSD(T)//M06-2X/aug-cc-pVTZ(-PP) method.

X	F		Cl		Br		I	
	E	H	E	H	E	H	E	H
**M = Cu**								
Cu^−^ + CH_3_X	0.0	0.0	0.0	0.0	0.0	0.0	0.0	0.0
RC	−6.6	−5.0	−8.0	−6.5	−8.7	−7.2	−9.8	−8.7
invTS	13.4	12.1	−2.9	−3.1	−6.9	−6.8	−9.8	−9.3
PC	4.7	3.4	−25.6	−25.9	−31.1	−31.0	−36.4	−35.9
XC	-	-	-	-	−1.8	−0.5	−11.8	−11.3
XTS	-	-	-	-	1.3	2.1	0.4	1.5
OxTS	33.3	31.7	24.3	23.5	19.3	18.8	14.3	14.1
MTS	4.6	2.7	−25.7	−26.5	-	-	-	-
HPC	−0.7	−2.5	-	-	-	-	-	-
HPTS	0.0	−2.1	-	-	−32.6	−33.0	−38.2	−38.3
**M = Ag**								
Ag^−^ + CH_3_X	0.0	0.0	0.0	0.0	0.0	0.0	0.0	0.0
RC	−6.4	−4.8	−7.7	−6.1	−8.3	−6.8	−9.3	−7.8
invTS	19.6	18.2	−0.1	−0.4	−4.6	−4.6	−8.5	−8.3
PC	17.9	16.8	−11.5	−11.6	−17.0	−16.7	−22.9	−22.4
XC	-	-	-	-	-1.4	0.0	−7.9	−6.9
XTS	-	-	-	-	1.1	2.4	0.3	1.5
OxTS	42.0	40.4	23.7	22.8	18.2	17.7	13.6	13.3
MTS	17.9	16.3	-	-	-	-	-	-
HPC	13.3	13.2	-	-	-	-	-	-
HPTS	16.1	14.1	−13.8	−14.5	−17.7	−18.1	−24.6	−24.6
**M = Au**								
Au^−^ + CH_3_X	0.0	0.0	0.0	0.0	0.0	0.0	0.0	0.0
RC	−8.6	−7.0	−10.0	−8.4	−10.6	−9.0	−11.1	−9.6
invTS	14.2	13.6	−2.7	−2.5	−6.7	−6.3	−9.6	−9.1
PC	12.5	12.0	−15.1	−14.5	−20.2	−19.3	−25.6	−24.5
XC	-	-	-	-	−3.3	−1.8	−11.0	−10.0
XTS	-	-	-	-	0.7	1.9	−0.3	0.8
OxTS	48.2	47.2	36.2	35.8	31.1	31.0	27.2	27.1
MTS	12.9	11.7	−14.7	−14.8	-	-	-	-
HPC	10.3	9.0	−14.7	−14.2	-	-	-	-
HPTS	13.1	11.7	−14.0	−14.1	−19.2	−18.9	−24.4	−23.8

## Supplementary Materials

The following are available online, Computational Methods, Figure S1: Natural orbitals of the singlet Cu^−^ anion obtained from the CASSCF calculation with an active space of (12e, 9o). Figure S2: The potential energy profile of the M^−^ + CH_3_Cl reaction, Figure S3: The potential energy profile of the M^−^ + CH_3_Br reaction, Figure S4: Structures of the selected stationary points along the potential energy profiles of Cu^−^ + CH_3_X (X = Cl, I) reactions, Figure S5: The intrinsic reaction coordinates (IRC) path scan of the oxidation addition transition state (OxTS) of a Cu^–^ + CH_3_F system. Table S1: Calculated reaction energies and enthalpies of M^−^ + CH_3_X reactions by M06-2X functionals, Table S2: Calculated electronic energies and enthalpies of the stationary points on the potential energy profiles of M^−^ + CH_3_X reactions with the M06-2X/aug-cc-pVTZ(-PP) method, Table S3: Natural population analysis (NPA) charges for stationary points of M^–^ + CH_3_X reactions, Table S4: T_1_ diagnostic of selected stationary points calculated by the CCSD(T) method, Table S5: Bond metrics for stationary points on the back-side attack pathway of M^−^ + CH_3_X reactions, Table S6: Imaginary vibrational frequencies of transition states, Table S7: Energetic comparison of selected transition states of Pd + CH_3_Cl reactions at different level of theories, Table S8: The charge transfer stabilization energy between a lone pair of nucleophiles M^−^ and C-X antibonding the *σ** orbital of halogen-bonded complexes [CH_3_–X∙∙∙M]^−^ as calculated under the NBO scheme, X = Br, I, Table S9: Orbital composition analysis of the C–M bond and X-M bond in the oxidative addition complex [CH_3_–M–X]^–^ under the NBO scheme, Table S10: Harmonic vibrational frequencies; coordinates of all computed structures.

## References

[1] Crabtree R.H. (2009). Organometallic Chemistry of the Transition Metals.

[2] Labinger J.A., Bercaw J.E. (2002). Understanding and exploiting C-H bond activation. Nature.

[3] Dupont J., Consorti C.S., Spencer J. (2005). The Potential of Palladacycles: More than Just Precatalysts. Chem. Rev..

[4] Pud De Phatt R.J. (2001). Platinum(IV) hydride chemistry. Coord. Chem. Rev..

[5] Puddephatt R.J. (2002). Coordinative Unsaturation in Platinum(IV) Chemistry: From Proposed Reaction Intermediates to the First Structurally Characterized Complexes. Angew. Chem. Int. Ed..

[6] Rendina L.M., Puddephatt R.J. (1997). Oxidative Addition Reactions of Organoplatinum(II) Complexes with Nitrogen-Donor Ligands. Chem. Rev..

[7] Crespo M., Martinez M., Nabavizadeh S.M., Rashidi M. (2014). Kinetico-mechanistic studies on C-X (X = H, F, Cl, Br, I) bond activation reactions on organoplatinum(II) complexes. Coord. Chem. Rev..

[8] Diefenbach A., de Jong G.T., Bickelhaupt F.M. (2005). Activation of H-H, C-H, C-C and C-Cl bonds by Pd and PdCl−. Understanding anion assistance in C-X bond activation. J. Chem. Theory Comput..

[9] de Jong G.T., Bickelhaupt F.M. (2007). Catalytic carbon−halogen bond activation: Trends in reactivity, selectivity, and solvation. J. Chem. Theory Comput..

[10] Hartwig J.F. (1998). Transition metal catalyzed synthesis of arylamines and aryl ethers from aryl halides and triflates: Scope and mechanism. Angew. Chem. Int. Ed..

[11] Martin R., Buchwald S.L. (2008). Palladium-catalyzed Suzuki-Miyaura cross-coupling reactions employing dialkylbiaryl phosphine ligands. Acc. Chem. Res..

[12] Barder T.E., Walker S.D., Martinelli J.R., Buchwald S.L. (2005). Catalysts for Suzuki-Miyaura coupling processes: Scope and studies of the effect of ligand structure. J. Am. Chem. Soc..

[13] Cundari T.R., Vaddadi S. (2004). Carbon-hydrogen and carbon-heteroatom bond activation using iridium (I) complexes. Inorg. Chim. Acta.

[14] Chowdhury A.K., Wilkins C.L. (1987). Reactions of atomic gold ions with aliphatic and aromatic hydrocarbons and alkyl halides. J. Am. Chem. Soc..

[15] Muramatsu S., Tsukuda T. (2019). Reductive Activation of Small Molecules by Anionic Coinage Metal Atoms and Clusters in the Gas Phase. Chem. Asian J..

[16] Liu J.C., Wang Y.G., Li J. (2017). Toward rational design of oxide-supported single-atom catalysts: Atomic dispersion of gold on ceria. J. Am. Chem. Soc..

[17] Grisel R., Weststrate K.J., Gluhoi A., Nieuwenhuys B.E. (2002). Catalysis by gold nanoparticles. Gold Bull..

[18] Mitsudome T., Kaneda K. (2013). Gold nanoparticle catalysts for selective hydrogenations. Green Chem..

[19] Muramatsu S., Koyasu K., Tsukuda T. (2016). Oxidative Addition of CH_3_I to Au^−^ in the Gas Phase. J. Phys. Chem. A.

[20] Chen Q., Zhao Y.X., Jiang L.X., Chen J.J., He S.G. (2018). Coupling of methane and carbon dioxide mediated by diatomic copper boride cations. Angew. Chem. Int. Ed..

[21] Wester R. (2021). Fifty years of nucleophilic substitution in the gas phase. Mass Spectrom. Rev..

[22] Ma J.B., Xu L.L., Liu Q.Y., He S.G. (2016). Activation of methane and ethane as mediated by the triatomic anion HNbN^−^: Electronic structure similarity with a Pt atom. Angew. Chem. Int. Ed..

[23] Wang W., Feng W., Wang W., Li P. (2018). Ab initio molecular dynamics simulation study on the stereo reactions between atomic oxygen anion and methane. Molecules.

[24] Xie J., Hase W.L. (2016). Rethinking the S_N_2 reaction. Science.

[25] Szabó I., Czakó G. (2015). Revealing a double-inversion mechanism for the F^−^ + CH_3_Cl S_N_2 reaction. Nat. Commun..

[26] Rijs N.J., González-Navarrete P., Schlangen M., Schwarz H. (2016). Penetrating the Elusive Mechanism of Copper-Mediated Fluoromethylation in the Presence of Oxygen through the Gas-Phase Reactivity of Well-Defined [LCuO]^+^ Complexes with Fluoromethanes (CH_4–n_F_n_, n= 1–3). J. Am. Chem. Soc..

[27] Meyer J., Tajti V., Carrascosa E., Győri T., Stei M., Michaelsen T., Bastian B., Czakó G., Wester R. (2021). Atomistic dynamics of elimination and nucleophilic substitution disentangled for the F^−^ + CH_3_CH_2_Cl reaction. Nat. Chem..

[28] Stei M., Carrascosa E., Kainz M.A., Kelkar A.H., Meyer J., Szabó I., Czakó G., Wester R. (2016). Influence of the leaving group on the dynamics of a gas-phase S_N_2 reaction. Nat. Chem..

[29] Viggiano A.A., Ard S.G., Shuman N.S. (2021). Temperature and energy dependences of ion-molecule reactions: Studies inspired by Diethard Böhme. Mass Spectrom. Rev..

[30] Muramatsu S., Koyasu K., Tsukuda T. (2017). Formation of Grignard Reagent-like Complex [CH_3_–M–I]^−^ via Oxidative Addition of CH_3_I on Coinage Metal Anions M^−^(M= Cu, Ag, Au) in the Gas Phase. Chem. Lett..

[31] Mikosch J., Trippel S., Eichhorn C., Otto R., Lourderaj U., Zhang J.X., Hase W.L., Weidemüller M., Wester R. (2008). Imaging nucleophilic substitution dynamics. Science.

[32] Xie J., Sun R., Siebert M.R., Otto R., Wester R., Hase W.L. (2013). Direct dynamics simulations of the product channels and atomistic mechanisms for the OH^–^ + CH_3_I reaction. Comparison with experiment. J. Phys. Chem. A.

[33] Zhang J.X., Mikosch J., Trippel S., Otto R., Weidemüller M., Wester R., Hase W.L. (2010). F− + CH3I → FCH3 + I− Reaction Dynamics. Nontraditional Atomistic Mechanisms and Formation of a Hydrogen-Bonded Complex. J. Phys. Chem. Lett..

[34] Purvis III G.D., Bartlett R.J. (1982). A full coupled-cluster singles and doubles model: The inclusion of disconnected triples. J. Chem. Phys..

[35] Pople J.A., Head-Gordon M., Raghavachari K. (1987). Quadratic configuration interaction. A general technique for determining electron correlation energies. J. Chem. Phys..

[36] Zhao Y., Truhlar D.G. (2008). The M06 suite of density functionals for main group thermochemistry, thermochemical kinetics, noncovalent interactions, excited states, and transition elements: Two new functionals and systematic testing of four M06-class functionals and 12 other functionals. Theor. Chem. Acc..

[37] Tasi D.A., Fábián Z., Czakó G. (2018). Benchmark ab Initio Characterization of the Inversion and Retention Pathways of the OH^–^ + CH_3_Y [Y= F, Cl, Br, I] S_N_2 Reactions. J. Phys. Chem. A.

[38] Lee C., Yang W., Parr R.G. (1988). Development of the Colle-Salvetti correlation-energy formula into a functional of the electron density. J. Phys. Rev. B.

[39] Tao J., Perdew J.P., Staroverov V.N., Scuseria G.E. (2003). Climbing the density functional ladder: Nonempirical meta-generalized gradient approximation designed for molecules and solids. J. Phys. Rev. Lett..

[40] Reed A.E., Curtiss L.A., Weinhold F. (1988). Intermolecular interactions from a natural bond orbital, donor-acceptor viewpoint. Chem. Rev..

[41] Ji X.Y., Zhao C.Y., Xie J. (2021). Investigating the role of halogen-bonded complexes in microsolvated Y^−^(H_2_O)_n_ + CH_3_I S_N_2 reactions. Phys. Chem. Chem. Phys..

[42] Lee T.J., Taylor P.R. (1989). A diagnostic for determining the quality of single-reference electron correlation methods. Int. J. Quantum Chem..

[43] Roos B.O., Taylor P.R., Sigbahn P.E.M. (1980). A complete active space SCF method (CASSCF) using a density matrix formulated super-CI approach. Chem. Phys..

[44] Feller D. (1996). The role of databases in support of computational chemistry calculations. J. Comput. Chem..

[45] Pritchard B.P., Altarawy D., Didier B., Gibson T.D., Windus T.L. (2019). New basis set exchange: An open, up-to-date resource for the molecular sciences community. J. Chem. Inf. Model..

[46] Roos B.O., Lindh R., Malmqvist P.Å., Veryazov V., Widmark P.O. (2005). New relativistic ANO basis sets for transition metal atoms. J. Phys. Chem. A.

[47] Schuchardt K.L., Didier B.T., Elsethagen T., Sun L., Gurumoorthi V., Chase J., Li J., Windus T.L. (2007). Basis set exchange: A community database for computational sciences. J. Chem. Inf. Model..

[48] Douglas M., Kroll N.M. (1974). Quantum electrodynamical corrections to the fine structure of helium. Ann. Phys..

[49] Hess B.A. (1985). Applicability of the no-pair equation with free-particle projection operators to atomic and molecular structure calculations. Phys. Rev. A.

[50] Hess B.A. (1986). Relativistic electronic-structure calculations employing a two-component no-pair formalism with external-field projection operators. Phys. Rev. A.

[51] Aquilante F., Autschbach J., Carlson R.K., Chibotaru L.F., Delcey M.G., De Vico L., Fdez. Galván I., Ferré N., Frutos L.M., Gagliardi L. (2016). Molcas 8: New capabilities for multiconfigurational quantum chemical calculations across the periodic table. J. Comput. Chem..

[52] Frisch M.J., Trucks G.W., Schlegel H.B., Scuseria G.E., Robb M.A., Cheeseman J.R., Scalmani G., Barone V., Mennucci B., Petersson G. (2013). Gaussian 09, Revision D.01.

[53] Bickelhaupt F.M., Ziegler T. (1995). Oxidative insertion as frontside S_N_2 substitution: A theoretical study of the model reaction system Pd + CH_3_Cl. Organometallics.

[54] Clark T., Hennemann M., Murray J.S., Politzer P. (2007). Halogen bonding: The σ-hole. J. Mol. Struct..

[55] Zhang X., Liu G., Ciborowski S., Bowen K. (2017). Stabilizing otherwise unstable anions with halogen bonding. Angew. Chem..

[56] Mallada B., Gallardo A., Lamanec M., de la Torre B., Špirko V., Hobza P., Jelinek P. (2021). Real-space imaging of anisotropic charge of σ-hole by means of Kelvin probe force microscopy. Science.

