# Supplementary material for: Computational Studies of Coinage Metal Anion M− + CH3X (X = F, Cl, Br, I) Reactions in Gas Phase
Source: Molecules. 2022 Jan 4;27(1):307. doi: 10.3390/molecules27010307 (PMC8746851; doi:10.3390/molecules27010307)
Supplement: Supplementary file 1 [file molecules-27-00307-s001.zip › molecules-1526131-supplementary.pdf]

Supporting Information

Computational Studies of Coinage Anion M^{-} + CH_3X (X=F, Cl, Br, I) Reactions in Gas Phase

Fan Wang, Xiaoyan Ji, Fei Ying, Jiatao Zhang, Chongyang Zhao, Jing Xie**

Key Laboratory of Cluster Science of Ministry of Education, School of Chemistry and Chemical Engineering, Beijing Institute of Technology, Beijing 100081, China

Corresponding Authors

Chongyang Zhao, e-mail: zhaochongyang@bit.edu.cn

Jing Xie, e-mail: jingxie@bit.edu.cn

Table of Contents

Table of Contents		S2
Computational Methods		S3
Figure S1	Natural orbitals of the singlet Cu^- anion obtained from CASSCF calculation	S4
Figure S2	Potential energy profile of M^- + CH_3F system	S5
Figure S3	Potential energy profile of M^- + CH_3Br system	S6
Figure S4	Structures of selected stationary points	S7
Figure S5	Intrinsic reaction coordinates of OxTS transition state	S8
Table S1	Reaction energies and enthalpies calculated by M06-2X functional	S9
Table S2	Electronic energies and enthalpies of stationary points calculated by M06-2X functional	S10
Table S3	NPA charges of stationary points	S11
Table S4	T_1 diagnostic of selected stationary points calculated by CCSD(T) method	S12
Table S5	Bond metrics of stationary points	S13
Table S6	Imaginary vibrational frequencies of transition states	S14
Table S7	Energetic comparison of selected transition states of Pd + CH_3Cl reaction at different level of theories	S14
Table S8	Charge transfer stabilization energy of halogen-bond interaction under NBO scheme	S15
Table S9	Composition of C–M/X–M bond in $[CH_3\text{–}M\text{–}X]^-$ under NBO scheme	S15
Table S10	Vibration frequencies of stationary points	S16
Cartesian coordinates of optimized structures		S20

Computational Methods

Structure optimization methods

The M06-2X/aug-cc-pVTZ(-PP) method was used to optimize most of the stationary points in this study. However, some stationary points were not converged using M06-2X functional, so different methods were used with the same basis sets to optimize these stationary points. Then the CCSD(T) energetics were reported on top of them. These following stationary points were localized with B3LYP functional [38]: HPC of Cu^- + CH_3F system, OxTS of Cu^- + CH_3Cl system, XTS and OxTS of Cu^- + CH_3Br system, OxTS of Cu^- + CH_3I system, HPC and OxTS of Ag^- + CH_3F system, and HPTS of Ag^- + CH_3Br system. TPSSh functional [39] was used for PC of Cu^- + CH_3F/CH_3Cl/CH_3Br system, and MTS of Cu^- + CH_3F.

CASSCF calculations

As shown in Table S4, the T1 diagnostic value of Cu^-, Ag^-, and Au^- anions are greater than 0.05, so ab initio calculations by means of the complete active space SCF method (CASSCF) were performed to characterize the singlet of Cu^-, Ag^-, and Au^- anions [43]. Relativistic basis sets of atomic natural orbital type of triple-ζ quality (ANO-RCC-VTZP) [44-47] were used for the metal atom. Scalar relativistic effects were included using the Douglas Kroll-Hess Hamiltonian [48-50]. We will use the case of Cu for illustration. The active space is CAS(12e, 9o), including 10 electrons from Cu 3d orbitals, 1 electron from Cu 4s orbital, and one extra electron. CASSCF calculations were performed using the MOLCAS-8.0 package [51].

According to CAS(12e, 9o) multireference calculation, the singlet of Cu^- anion is best described as having an electronic configuration of $d_1^2d_2^2d_3^2d_4^2d_5^2s^2$, as shown in Figure S1. The dominant configuration of singlet has a weight of 93%, indicating the singlet is nearly single reference. Calculations with extended orbitals CAS(12e, 12o) give similar multireference character of singlet Cu^- anion. In comparison, the triplet is 70.6 kcal/mol (CASSCF energy) higher in energy than singlet. Ag^- and Au^- anions are also single-reference, where the weight of the dominant configuration $d_1^2d_2^2d_3^2d_4^2d_5^2s^2$ is 94% and 93%.

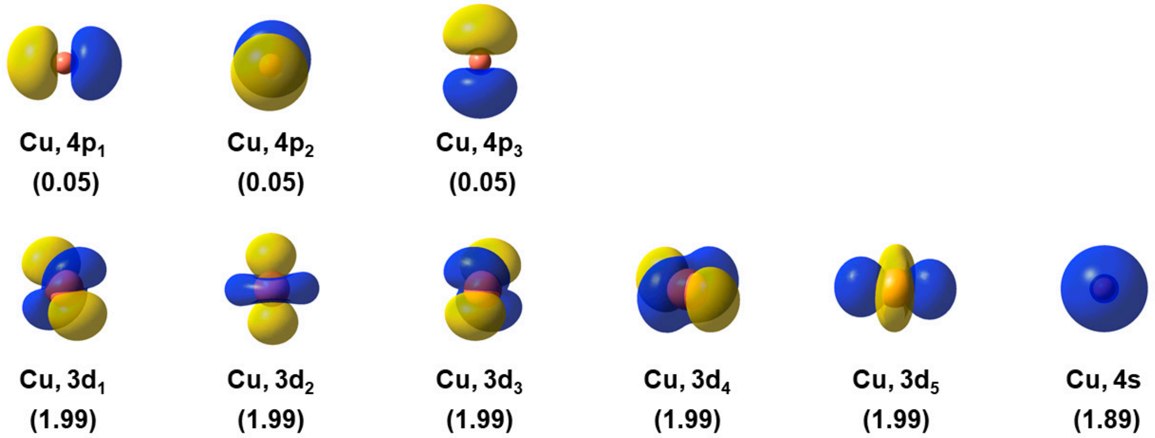

Figure S1. Natural orbitals of the singlet Cu^{-} anion obtained from CASSCF calculation with an active space of (12e, 9o). The numbers in the parentheses represent occupation number.

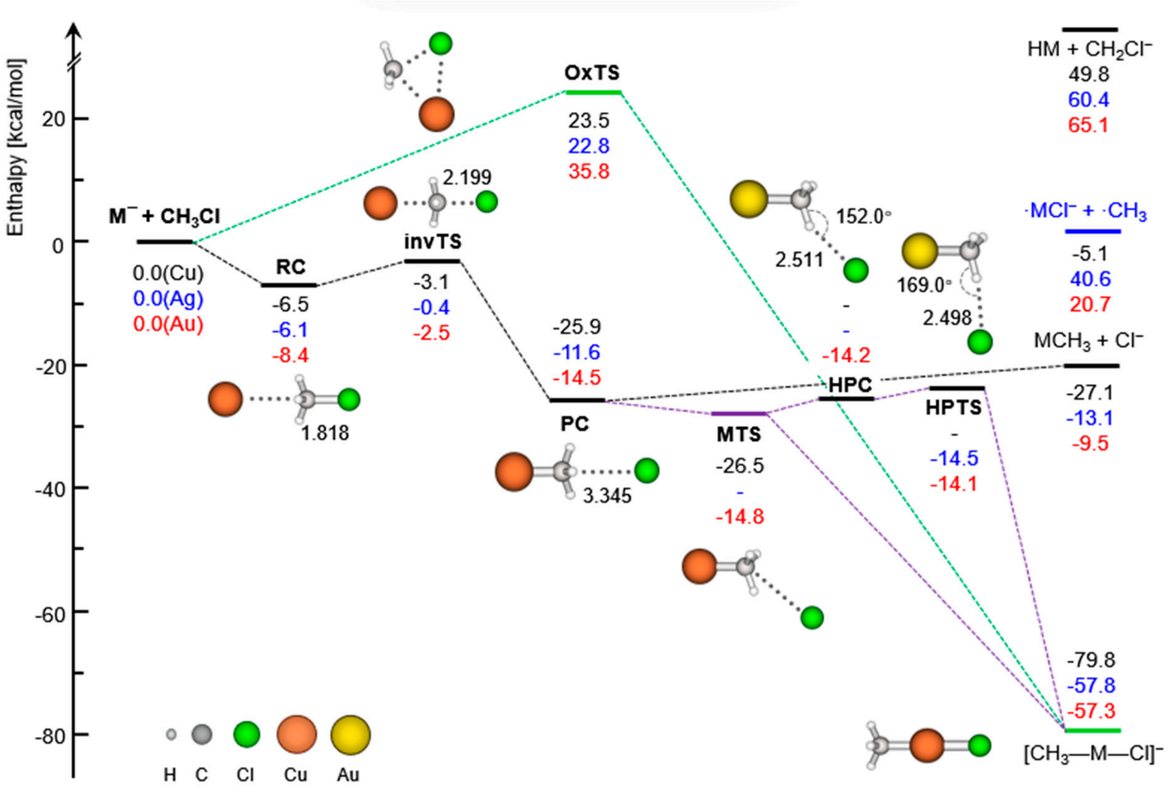

Figure S2. The potential energy profile of M^- + CH_3Cl reaction, M = Cu, Ag, Au.

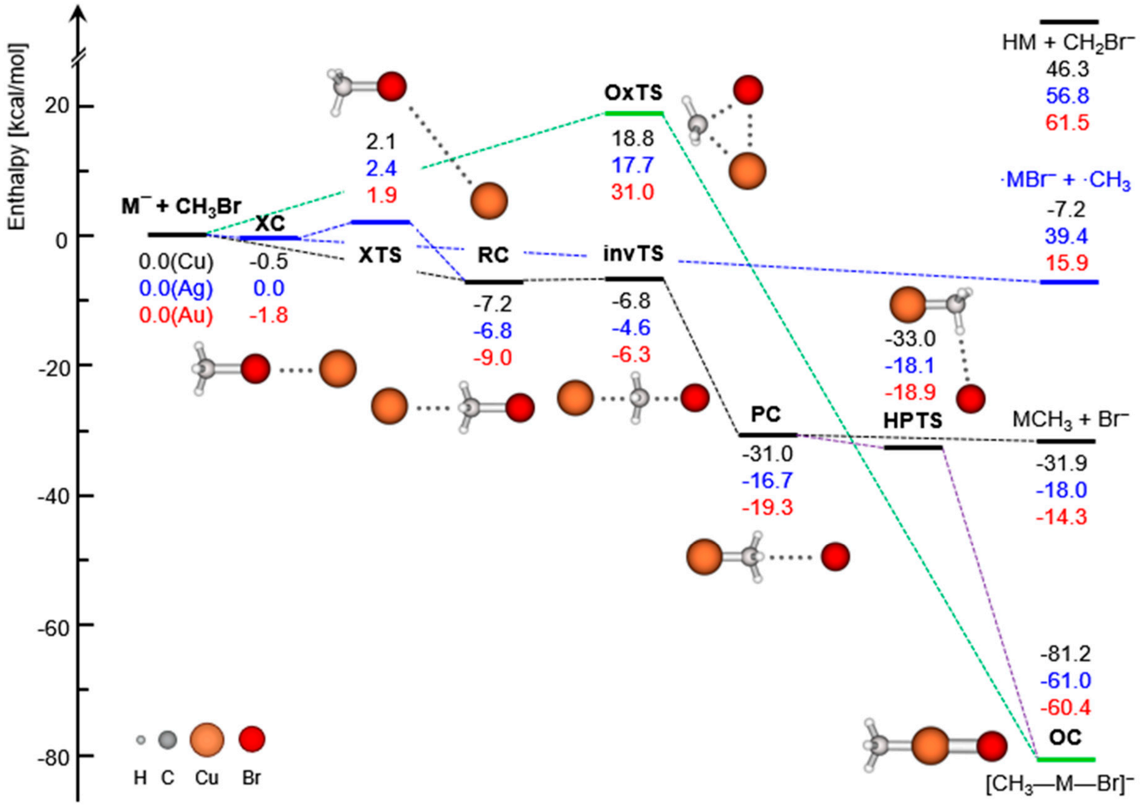

Figure S3. The potential energy profile of M⁻ + CH3Br reaction, M = Cu, Ag, Au.

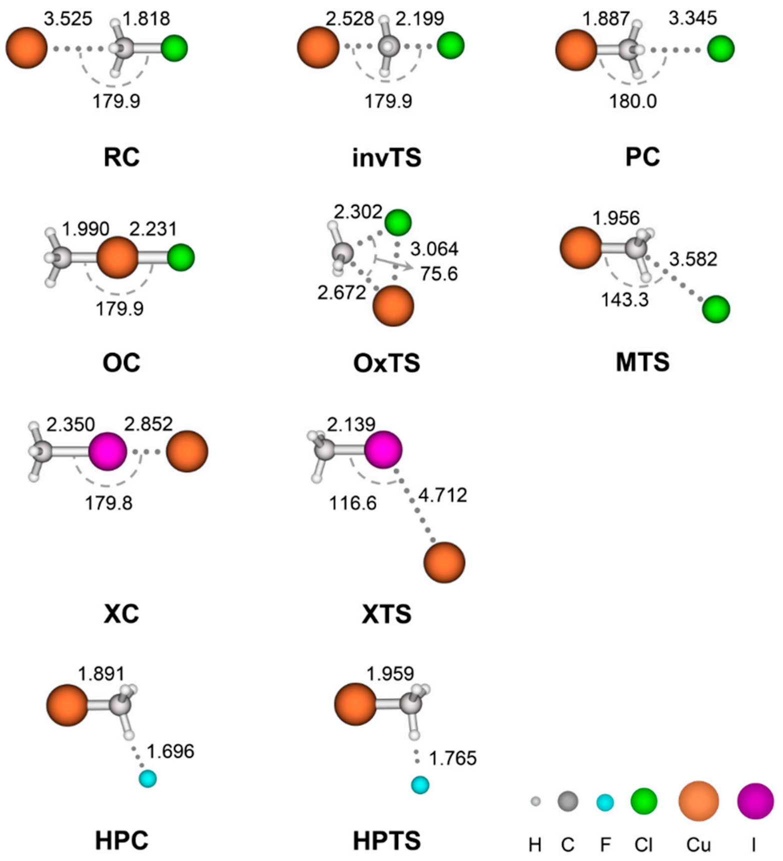

Figure S4. Structures of the selected stationary points along the potential energy profiles of Cu^{-} + CH_3X (X = Cl, I) reactions. Distances are in Angstrom and angles are in degree.

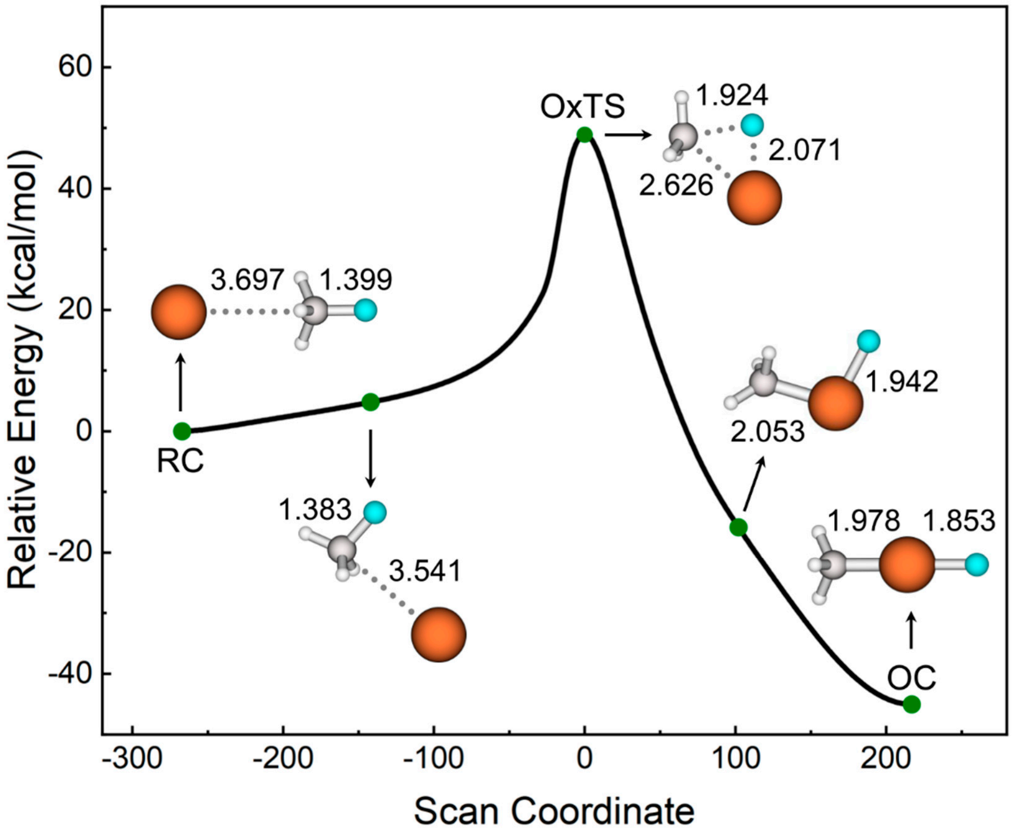

Figure S5. The intrinsic reaction coordinates (IRC) path scan of the oxidation addition transition state (OxTS) of Cu^{-} + CH_3F System. The distances are in angstrom. Color code: H, white; C, gray; F, light blue; Cu, orange.

Table S1. Calculated reaction energies (E) and enthalpies at 298.15 K (H) of M^{-} + CH_3X (M = Cu, Ag, Au; Y = F, Cl, Br, I) reactions with various product channels. Values (in kcal/mol) are calculated with M06-2X/aug-cc-pVTZ(-PP) method.

**Products**	**X**	**F**		**Cl**		**Br**		**I**	
		**E**	**H**	**E**	**H**	**E**	**H**	**E**	**H**
					**M = Cu**				
OI	$[CH_3\text{–}Cu\text{–}X]^{-}$	-50.7	-52.0	-70.8	-71.3	-75.0	-75.2	-78.1	-78.0
S_N2	$CuCH_3 + X^{-}$	17.4	14.4	-18.4	-20.4	-26.8	-28.4	-34.3	-35.7
XA	$CuX^{-} + CH_3$	20.2	17.1	-1.6	-3.9	-6.8	-8.8	-10.7	-12.5
PT	$HCu + CH_2X^{-}$	75.3	69.8	61.7	56.7	57.2	52.3	52.3	47.6
					**M = Ag**				
OI	$[CH_3\text{–}Ag\text{–}X]^{-}$	-29.1	-30.4	-53.1	-53.6	-58.5	-58.7	-63.0	-62.9
S_N2	$AgCH_3 + X^{-}$	28.7	25.8	-7.1	-8.9	-15.4	-17.0	-23.0	-24.3
XA	$AgX^{-} + CH_3$	32.8	29.6	7.2	4.9	0.9	-1.1	-4.3	-6.0
PT	$HAg + CH_2X^{-}$	83.7	77.9	70.0	64.8	65.5	60.5	60.6	55.7
					**M = Au**				
OI	$[CH_3\text{–}Au\text{–}X]^{-}$	-34.2	-34.9	-56.2	-56.1	-61.2	-60.8	-65.4	-64.7
S_N2	$AuCH_3 + X^{-}$	25.5	23.3	-10.3	-11.5	-18.6	-19.5	-26.2	-26.8
XA	$AuX^{-} + CH_3$	42.4	39.2	16.2	13.9	9.4	7.4	3.3	1.6
PT	$HAu + CH_2X^{-}$	79.4	74.7	65.8	61.6	61.3	57.2	56.4	52.5

Table S2. Calculated electronic energies (E) and enthalpies at 298.15 K (H) (kcal/mol) of the stationary points on the potential energy profiles of M^- + CH_3X reactions with M06-2X/aug-cc-pVTZ(-PP) method. Values that are optimized by methods other than M06-2X functional are labeled.

X	F		Cl		Br		I	
	E	H	E	H	E	H	E	H
				**M = Cu**				
Cu^- + CH_3X	0.0	0.0	0.0	0.0	0.0	0.0	0.0	0.0
RC	-5.6	-4.0	-7.1	-5.6	-8.1	-6.7	-10.0	-8.9
invTS	23.7	22.5	0.7	0.5	-5.6	-5.5	-	-9.5
PC	18.2[a]	16.9[a]	-15.7[a]	-16.0[a]	-24.1[a]	-24.0[a]	-32.4	-32.0
XC	-	-	-	-	-1.3	0.0	-7.8	-7.3
XTS	-	-	-	-	1.2[b]	2.0[b]	0.7	1.9
OxTS	43.3	41.7	31.3[b]	30.6[b]	24.9[b]	24.4[b]	21.4[b]	21.2[b]
MTS	18.2[a]	16.2[a]	-17.2	-18.0	-	-	-	-
HPC	13.1[b]	11.3[b]	-	-	-	-	-	-
HPTS	12.0	9.9	-	-	-27.2	-27.6	-34.3	-34.4
				**M = Ag**				
Ag^- + CH_3X	0.0	0.0	0.0	0.0	0.0	0.0	0.0	0.0
RC	-5.5	-3.9	-6.9	-5.3	-7.8	-6.3	-9.3	-7.9
invTS	29.3	27.9	4.1	3.8	-2.8	-2.8	-8.0	-7.8
PC	27.5	26.4	-5.8	-5.9	-14.0	-13.8	-21.5	-20.9
XC	-	-	-	-	-0.9	0.5	-5.6	-4.6
XTS	-	-	-	-	1.4[c]	2.7[c]	0.7	1.9
OxTS	50.7[b]	49.0[b]	33.7	32.8	27.0	26.5	23.4	23.2
MTS	27.5	25.8	-	-	-	-	-	-
HPC	23.5[b]	23.5[b]	-	-	-	-	-	-
HPTS	26.0	24.0	-8.3	-9.0	-14.1[b]	-14.5[b]	-23.2	-23.3
				**M = Au**				
Au^- + CH_3X	0.0	0.0	0.0	0.0	0.0	0.0	0.0	0.0
RC	-7.5	-5.9	-9.0	-7.4	-9.7	-8.2	-9.3	-7.7
invTS	22.1	21.4	0.4	0.6	-5.1	-4.7	-9.0	-8.5
PC	16.3	15.8	-15.2	-14.7	-23.0	-22.1	-29.9	-28.8
XC	-	-	-	-	-2.2	-0.8	-7.7	-6.7
XTS	-	-	-	-	1.2	2.4	0.5	1.6
OxTS	55.2	54.2	38.3	37.9	31.7	31.6	27.5	27.5
MTS	18.0	16.7	-13.3	-13.4	-	-	-	-
HPC	14.3	13.0	-14.6	-14.1	-	-	-	-
HPTS	15.6	14.1	-14.5	-14.6	-22.0	-21.7	-28.8	-28.2

[a] M06-2X//TPSSh/aug-cc-pVTZ(-PP) method was used.
[b] M06-2X//B3LYP/aug-cc-pVTZ(-PP) method was used.
[c] M06-2X//MP2/aug-cc-pVTZ(-PP) method was used.

Table S3. Natural population analysis (NPA) charges for stationary points of M^- + CH_3X (M = Cu, Ag, Au; X = F, Cl, Br, I) reactions.

	XC			XTS			RC			invTS			PC		
X	q(C)	q(X)	q(M)	q(C)	q(X)	q(M)	q(C)	q(X)	q(M)	q(C)	q(X)	q(M)	q(C)	q(X)	q(M)
M=Cu															
F	-	-	-	-	-	-	-0.105	-0.415	-0.980	-0.457	-0.760	-0.332	-0.855	-0.941	0.156
Cl	-	-	-	-	-	-	-0.527	-0.159	-0.957	-0.538	-0.523	-0.531	-0.966	-0.968	0.292
Br	-0.671	-0.036	-0.857	-0.617	-0.001	-0.997	-0.585	-0.153	-0.910	-0.589	-0.433	-0.582	-0.983	-0.967	0.311
I	-0.839	-0.178	-0.507	-0.734	0.102	-0.992	-0.664	-0.225	-0.746	-0.663	-0.250	-0.717	-0.995	-0.975	0.344
M=Ag															
F	-	-	-	-	-	-	-0.104	-0.415	-0.981	-0.475	-0.804	-0.283	-0.746	-0.931	0.066
Cl	-	-	-	-	-	-	-0.527	-0.155	-0.962	-0.534	-0.600	-0.452	-0.882	-0.959	0.227
Br	-0.663	-0.016	-0.893	-0.638	0.021	-0.944	-0.584	-0.141	-0.926	-0.580	-0.524	-0.490	-0.902	-0.957	0.246
I	-0.804	-0.036	-0.716	-0.734	0.103	-0.994	-0.667	-0.147	-0.836	-0.647	-0.418	-0.543	-0.930	-0.965	0.284
M=Au															
F	-	-	-	-	-	-	-0.105	-0.422	-0.979	-0.369	-0.759	-0.435	-0.639	-0.933	-0.068
Cl	-	-	-	-	-	-	-0.518	-0.167	-0.963	-0.468	-0.527	-0.604	-0.719	-0.950	0.027
Br	-0.668	0.006	-0.912	-0.633	0.024	-0.998	-0.571	-0.146	-0.939	-0.521	-0.455	-0.634	-0.734	-0.947	0.041
I	-0.823	0.011	-0.744	-0.998	0.112	-0.735	-0.641	-0.125	-0.893	-0.592	-0.371	-0.658	-0.749	-0.946	0.059
	**MTS**			**HPC**			**HPTS**			**OxTS**			**OC**		
X	q(C)	q(X)	q(M)	q(C)	q(X)	q(M)	q(C)	q(X)	q(M)	q(C)	q(X)	q(M)	q(C)	q(X)	q(M)
M=Cu															
F	-0.865	-0.944	0.166	-1.014	-0.940	0.288	-1.014	-0.953	0.315	-0.483	-0.719	-0.287	-1.216	-0.854	0.548
Cl	-0.985	-0.981	0.330	-	-	-	-	-	-	-0.558	-0.527	-0.451	-1.207	-0.761	0.434
Br	-	-	-	-	-	-	-1.047	-0.987	0.410	-0.601	-0.494	-0.441	-1.206	-0.738	0.407
I	-	-	-	-	-	-	-1.053	-0.989	0.423	-0.635	-0.488	-0.410	-1.209	-0.710	0.379
M=Ag															
F	-0.763	-0.937	0.085	-0.925	-0.936	0.215	-0.860	-0.949	0.168	-0.440	-0.706	-0.339	-1.173	-0.872	0.525
Cl	-	-	-	-	-	-	-0.987	-0.984	0.355	-0.562	-0.575	-0.391	-1.168	-0.793	0.430
Br	-	-	-	-	-	-	-0.930	-0.976	0.286	-0.603	-0.529	-0.399	-1.167	-0.768	0.403
I	-	-	-	-	-	-	-1.001	-0.988	0.383	-0.633	-0.484	-0.418	-1.169	-0.739	0.372
M=Au															
F	-0.677	-0.945	-0.032	-0.775	-0.922	0.033	-0.805	-0.954	0.115	-0.347	-0.644	-0.481	-1.036	-0.827	0.343
Cl	-0.755	-0.973	0.080	-0.764	-0.975	0.093	-0.793	-0.980	0.138	-0.485	-0.539	-0.516	-1.041	-0.736	0.240
Br	-	-	-	-	-	-	-0.792	-0.982	0.143	-0.525	-0.500	-0.521	-1.041	-0.711	0.211
I	-	-	-	-	-	-	-0.794	-0.986	0.151	-0.561	-0.456	-0.531	-1.045	-0.676	0.176

Table S4. T_1 diagnostic of selected stationary points calculated by CCSD(T) method.

M^-	Cu^-	Ag^-	Au^-
	0.051	0.051	0.036

	RC	invTS	PC	OxTS	OC
Au^- + CH_3F	0.026	0.022	0.021	0.028	0.016
Au^- + CH_3Cl	0.025	0.023	0.018	0.030	0.015
Au^- + CH_3Br	0.022	0.020	0.017	0.025	0.014
Au^- + CH_3I	0.022	0.021	0.017	0.026	0.014

Table S5. Bond metrics for stationary points on back-side attack pathway of M^- + CH_3X (M = Cu, Ag, Au; X = F, Cl, Br, I) reactions. Bond distances are in Angstrom.

	F				Cl				Br				I			
	C−X	C−M	M−X	M−C−X	C−X	C−M	M−X	M−C−X	C−X	C−M	M−X	M−C−X	C−X	C−M	M−X	M−C−X
CH_3X	1.380	-	-	-	1.783	-	-	-	1.941	-	-	-	2.140	-	-	-
**M=Cu**																
RC	1.399	3.697	5.096	179.2	1.818	3.525	5.343	179.9	2.002	3.343	5.345	179.0	2.295	2.968	5.263	179.8
invTS	1.978	2.259	4.238	180.0	2.199	2.528	4.726	179.9	2.268	2.628	4.896	179.8	-	-	-	-
PC	2.562	1.904	4.466	180.0	3.345	1.887	5.232	180.0	3.538	1.887	5.425	179.9	3.914	1.958	5.794	160.1
XC	-	-	-	-	-	-	-	-	1.989	5.309	3.320	0.2	2.350	5.202	2.852	0.1
XTS	-	-	-	-	-	-	-	-	1.963	7.445	6.029	38.3	2.139	5.982	4.712	44.8
OxTS	1.924	2.626	2.071	51.36	2.302	2.672	3.064	75.6	2.446	2.742	3.274	78.0	2.682	2.858	3.668	82.8
OC	3.831	1.978	1.853	0.0	4.221	1.990	2.231	0.0	4.365	1.993	2.372	0.0	4.552	1.996	2.556	0.0
CH_3M	-	1.958	-	-	-	1.958	-	-	-	1.958	-	-	-	1.958	-	-
**M=Ag**																
RC	1.399	3.745	5.143	179.3	1.815	3.613	5.428	179.0	1.994	3.467	5.461	179.0	2.235	3.229	5.464	179.1
invTS	2.096	2.345	4.441	179.9	2.311	2.563	4.874	179.8	2.387	2.633	5.020	179.9	2.486	2.734	5.220	179.4
PC	2.560	2.162	4.722	179.0	3.299	2.140	5.439	178.8	3.474	2.140	5.611	175.9	3.793	2.137	5.889	165.9
XC	-	-	-	-	-	-	-	-	1.976	5.481	3.506	0.2	2.242	5.506	3.264	0.3
XTS	-	-	-	-	-	-	-	-	1.921	5.651	4.358	40.0	2.138	6.076	4.793	44.5
OxTS	1.955	2.678	2.471	62.2	2.369	2.901	2.901	65.9	2.494	2.957	3.062	67.7	2.681	2.989	3.464	75.1
OC	4.209	2.132	2.077	0.0	4.600	2.150	2.450	0.0	4.733	2.156	2.577	0.1	4.910	2.162	2.748	0.0
CH_3M	-	2.136	-	-	-	2.136	-	-	-	2.136	-	-	-	2.136	-	-
**M=Au**																
RC	1.403	3.467	4.870	178.6	1.820	3.376	5.195	178.8	1.993	3.291	5.283	179.2	2.217	3.191	5.408	179.7
invTS	1.957	2.269	4.226	180.0	2.197	2.528	4.724	179.9	2.290	2.602	4.892	180.0	2.430	2.693	5.123	179.7
PC	2.500	2.038	4.538	179.1	3.148	2.022	5.170	178.9	3.319	2.021	5.340	179.4	3.538	2.020	5.557	179.0
XC	-	-	-	-	-	-	-	-	1.968	5.297	3.329	0.2	2.234	5.266	3.032	0.1
XTS	-	-	-	-	-	-	-	-	1.937	5.767	4.441	39.3	2.136	5.468	4.294	46.7
OxTS	1.806	2.403	2.605	75.0	2.329	2.590	3.136	79.0	2.474	2.633	3.284	80.0	2.668	2.692	3.519	82.1
OC	4.088	2.016	2.072	0.0	4.467	2.036	2.431	0.0	4.602	2.042	2.561	0.1	4.771	2.050	2.721	0.0
CH_3M	-	2.008	-	-	-	2.008	-	-	-	2.008	-	-	-	2.008	-	-

Table S6. Imaginary vibrational frequencies of transition states.

	Imaginary vibrational frequencies (cm^{-1})			
	F	Cl	Br	I
**M=Cu**				
invTS	i501	i413	i320	i31[a]
OxTS	i538	i429	i371	i326
XTS	-	-	i28	i65
MTS	i58	i41	-	-
HPTS	i44	-	i46	i31
**M=Ag**				
invTS	i391	i398	i331	i275
OxTS	i470	i419	i348	i334
XTS	-	-	i52	i63
MTS	i41	-	-	-
HPTS	i51	i38	i42	i26
**M=Au**				
invTS	i481	i438	i387	i334
OxTS	i606	i532	i450	i448
XTS	-	-	i38	i66
MTS	i94	i29	-	-
HPTS	i76	i19	i17	i29

[a]We note that imaginary frequency of invTS of Cu^{-} + CH_3I reaction, being i31 cm^{-1}, represents a half umbrella-inversion of CH_3-group, is too low, and attempts to search for the correct invTS were not successful using multiple DFT and basis sets.

Table S7. Energetic comparison of selected transition states of Pd + CH_3Cl reaction at different level of theories.

	Bickelhaupt [37]		This work			
Method	LDA	NL-SCF	M06-2X/aug-cc-pVTZ-PP		CCSD(T)//M06-2X/aug-cc-pVTZ-PP	
(kcal/mol)	E	E	E	H (298.15 K)	E	H (298.15 K)
Pd + CH_3Cl	0	0	0	0	0	0
TS(S_N2/Cl-ra)	22.9	29.6	48.4	47.5	40.5	39.5
OxTS	-16.1	1.7	10.5	10.4	1.7	1.5

Table S8. The charge transfer stabilization energy between lone pair of nucleophile M^- and C–X antibonding σ^* orbital of the halogen-bonded complexes $[CH_3\text{–}X\cdots M]^-$ as calculated under NBO scheme. X = Br, I.

	$E_{stabilization}$ (kcal/mol)	
M	X = Br	X = I
Cu	10.0	56.6
Ag	7.4	21.7
Au	8.3	31.8

Table S9. Orbital composition analysis of the C–M bond and X–M bond in the oxidative addition complex $[CH_3\text{–}M\text{–}X]^-$ (M = Cu, Ag, Au; X = F, Cl, Br, I) under the NBO scheme.

		C-M bond				X-M bond			
M	X	C		M		X		M	
Cu	F	86%	($sp^{2.95}$)	14%	($sp^{0.87}d^{0.12}$)	95%	($sp^{1.65}$)	5%	($sp^{1.41}d^{0.17}$)
	Cl	85%	($sp^{2.85}$)	15%	($sp^{0.71}d^{0.09}$)	91%	($sp^{2.07}$)	9%	($sp^{1.55}d^{0.10}$)
	Br	85%	($sp^{2.85}$)	15%	($sp^{0.68}d^{0.08}$)	90%	($sp^{2.64}$)	10%	($sp^{1.60}d^{0.09}$)
	I	85%	($sp^{2.83}$)	15%	($sp^{0.64}d^{0.07}$)	89%	($sp^{2.86}$)	11%	($sp^{1.65}d^{0.07}$)
Ag	F	85%	($sp^{3.52}$)	15%	($sp^{0.89}d^{0.12}$)	95%	($sp^{2.61}$)	5%	($sp^{1.26}d^{0.13}$)
	Cl	84%	($sp^{3.42}$)	16%	($sp^{0.74}d^{0.09}$)	92%	($sp^{2.93}$)	8%	($sp^{1.44}d^{0.09}$)
	Br	84%	($sp^{3.41}$)	16%	($sp^{0.71}d^{0.09}$)	91%	($sp^{3.58}$)	9%	($sp^{1.50}d^{0.08}$)
	I	83%	($sp^{3.38}$)	17%	($sp^{0.67}d^{0.08}$)	90%	($sp^{3.75}$)	10%	($sp^{1.57}d^{0.07}$)
Au	F	72%	($sp^{3.17}$)	28%	($sp^{0.04}d^{0.30}$)	-	-	-	-
	Cl	78%	($sp^{3.22}$)	22%	($sp^{0.84}d^{0.25}$)	90%	($sp^{3.58}$)	10%	($sp^{1.57}d^{0.25}$)
	Br	78%	($sp^{3.26}$)	22%	($sp^{0.79}d^{0.23}$)	89%	($sp^{4.35}$)	11%	($sp^{1.65}d^{0.23}$)
	I	78%	($sp^{3.27}$)	22%	($sp^{0.74}d^{0.22}$)	88%	($sp^{4.45}$)	12%	($sp^{1.71}d^{0.20}$)

Table S10. Harmonic vibrational frequencies (in cm^{-1}) given by M06-2X/aug-cc-pVTZ(-PP) method.

Reactants / Products	CH_3F	CH_3Cl	CH_3Br	CH_3F	$CuCH_3$	$AgCH_3$	$AuCH_3$	CH_3
1	1109	769	637	577	500	447	561	525
2	1208	1031	974	908	635	652	806	1416
3	1208	1031	974	908	635	652	806	1416
4	1495	1384	1336	1286	1101	1089	1213	3132
5	1507	1490	1483	1477	1451	1454	1460	3309
6	1507	1490	1483	1477	1451	1454	1460	3309
7	3064	3090	3097	3101	3054	3070	3066	
8	3149	3189	3205	3214	3151	3172	3168	
9	3149	3189	3205	3214	3151	3172	3169	

Reactants / Products	CH_2F^-	CH_2Cl^-	CH_2Br^-	CH_2I^-	HCu	HAg	HAu
1	783	493	424	365	1732	1610	2287
2	1112	1029	1006	959			
3	1214	1067	1006	972			
4	1428	1394	1380	1374			
5	2858	2934	2936	2940			
6	2908	2993	2996	3001			

$Cu^- + CH_3F$	XC	XTS	RC	invTS	PC	MTS	HPC	HPTS	OxTS	OC	MX^-
1	-	-	53	i501	48	i58	62	i44	i538	153	457
2			63	185	48	66	210	194	114	157	
3			67	187	129	129	288	261	123	445	
4			1033	187	499	503	533	473	326	542	
5			1173	818	610	583	703	653	577	569	
6			1178	824	610	628	764	727	718	583	
7			1458	884	1036	1042	1195	1120	963	1115	
8			1494	1391	1421	1421	1417	1420	1424	1454	
9			1496	1396	1421	1422	1467	1479	1441	1458	
10			3074	3151	3063	3057	2491	2673	3134	2989	
11			3172	3334	3171	3160	3026	3104	3273	3045	
12			3174	3335	3171	3170	3075	3158	3278	3054	

$Cu^- + CH_3Cl$	XC	XTS	RC	invTS	PC	MTS	HPC	HPTS	OxTS	OC	MX^-
1	-	-	47	i413	7	i41	-	-	i429	113	278
2			50	137	10	66			78	117	
3			54	148	56	100			134	285	
4			637	151	527	480			167	479	
5			994	837	633	626			593	568	
6			1001	848	633	641			712	580	
7			1338	904	1106	1100			1012	1114	
8			1472	1410	1435	1435			1429	1455	
9			1473	1416	1435	1445			1432	1459	
10			3099	3142	3044	3059			3115	2997	
11			3213	3323	3146	3161			3237	3057	
12			3218	3337	3146	3176			3257	3066	

Table S10 Continues

Cu^{-} + CH_3Br	XC	XTS	RC	invTS	PC	MTS	HPC	HPTS	OxTS	OC	MX^{-}
1	47	i28	52	i320	18	-	-	i46	i371	99	194
2	64	1	85	112	20			65	58	105	
3	66	18	88	129	40			77	125	207	
4	518	553	468	134	529			489	160	474	
5	900	948	935	828	640			654	581	561	
6	904	950	935	837	640			683	679	578	
7	1266	1314	1268	941	1112			1077	983	1110	
8	1476	1474	1461	1418	1437			1432	1428	1453	
9	1477	1477	1462	1428	1437			1450	1433	1457	
10	3082	3075	3106	3132	3040			3023	3118	2995	
11	3188	3174	3237	3308	3142			3147	3242	3057	
12	3190	3177	3241	3323	3142			3172	3260	3063	
**Cu^{-} + CH_3I**	**XC**	**XTS**	**RC**	**invTS**	**PC**	**MTS**	**HPC**	**HPTS**	**OxTS**	**OC**	**MX^{-}**
1	82	i65	25	i31	30	-	-	i31	i326	95	160
2	88	8	95	103	48			81	51	98	
3	89	115	103	111	90			92	120	167	
4	329	553	220	202	478			486	161	471	
5	678	896	820	826	624			638	552	572	
6	688	907	839	832	627			666	651	580	
7	1054	1277	1106	1084	1093			1075	954	1114	
8	1449	1477	1438	1439	1438			1437	1427	1453	
9	1451	1485	1442	1441	1443			1450	1434	1458	
10	3062	3102	3102	3111	3067			3048	3126	3000	
11	3188	3206	3256	3270	3168			3162	3259	3064	
12	3190	3218	3268	3279	3179			3178	3264	3071	
**Ag^{-} + CH_3F**	**XC**	**XTS**	**RC**	**invTS**	**PC**	**MTS**	**HPC**	**HPTS**	**OxTS**	**OC**	**MX^{-}**
1	-	-	50	i391	62	i41	57	i51	i470	133	361
2			54	163	64	78	217	158	69	135	
3			60	163	126	129	289	185	136	395	
4			1035	181	375	388	437	425	213	459	
5			1172	766	637	634	719	596	601	594	
6			1179	768	645	652	771	670	747	605	
7			1458	885	1009	1022	1176	1071	971	1104	
8			1494	1400	1424	1424	1424	1423	1417	1458	
9			1497	1401	1424	1426	1471	1453	1445	1459	
10			3074	3159	3125	3119	2514	2959	3112	3004	
11			3172	3335	3248	3240	3057	3125	3230	3070	
12			3173	3337	3252	3242	3111	3174	3260	3072	
**Ag^{-} + CH_3Cl**	**XC**	**XTS**	**RC**	**invTS**	**PC**	**MTS**	**HPC**	**HPTS**	**OxTS**	**OC**	**MX^{-}**
1	-	-	55	i398	30	-	-	i38	i419	103	230
2			64	126	33			108	34	106	
3			69	139	66			135	84	251	
4			651	144	409			427	170	428	
5			1006	821	641			660	533	587	
6			1009	823	643			710	664	602	
7			1347	845	1065			1073	938	1101	
8			1474	1403	1439			1436	1429	1458	
9			1476	1406	1439			1460	1434	1461	
10			3100	3155	3113			3031	3130	3014	
11			3214	3344	3224			3166	3271	3082	
12			3217	3348	3225			3194	3276	3087	

Table S10 Continues

**Ag^{-} + CH_3Br**	**XC**	**XTS**	**RC**	**invTS**	**PC**	**MTS**	**HPC**	**HPTS**	**OxTS**	**OC**	**MX^{-}**
1	41	i52	42	i331	13	-	-	i42	i348	90	146
2	63	18	74	99	21			41	71	95	
3	66	42	75	132	47			77	134	176	
4	551	655	489	138	411			435	157	423	
5	916	975	938	815	644			669	551	584	
6	919	978	939	817	645			693	658	599	
7	1281	1345	1274	847	1068			1102	932	1098	
8	1478	1499	1463	1409	1439			1436	1432	1457	
9	1479	1503	1464	1411	1440			1439	1440	1460	
10	3083	3103	3106	3145	3109			3047	3137	3015	
11	3187	3213	3236	3333	3219			3160	3273	3085	
12	3190	3214	3237	3337	3221			3166	3284	3090	

**Ag^{-} + CH_3I**	**XC**	**XTS**	**RC**	**invTS**	**PC**	**MTS**	**HPC**	**HPTS**	**OxTS**	**OC**	**MX^{-}**
1	44	i63	53	i275	33	-	-	i26	i334	87	115
2	70	8	91	84	44			34	49	91	
3	80	116	97	116	63			82	133	143	
4	385	557	348	119	431			434	175	419	
5	782	897	869	802	640			670	541	593	
6	788	908	875	810	649			706	649	602	
7	1163	1278	1189	888	1076			1071	948	1103	
8	1463	1478	1451	1418	1440			1436	1435	1456	
9	1465	1485	1451	1419	1444			1453	1441	1462	
10	3076	3102	3107	3129	3084			3054	3130	3020	
11	3192	3207	3252	3310	3195			3175	3267	3089	
12	3194	3218	3254	3318	3200			3190	3269	3100	

**Au^{-} + CH_3F**	**XC**	**XTS**	**RC**	**invTS**	**PC**	**MTS**	**HPC**	**HPTS**	**OxTS**	**OC**	**MX^{-}**
1	-	-	62	i481	75	i94	68	i76	i606	161	356
2			78	209	79	95	225	180	14	164	
3			88	219	153	153	312	222	205	443	
4			1019	219	497	524	543	549	337	563	
5			1166	940	756	702	839	814	861	765	
6			1169	941	763	791	867	874	927	773	
7			1447	1041	1124	1151	1289	1177	1201	1222	
8			1490	1393	1424	1427	1435	1416	1439	1466	
9			1495	1393	1424	1434	1494	1484	1493	1472	
10			3082	3160	3112	3092	2305	2820	3025	2998	
11			3181	3341	3228	3201	3076	3093	3141	3049	
12			3182	3342	3233	3205	3134	3136	3217	3061	

**Au^{-} + CH_3Cl**	**XC**	**XTS**	**RC**	**invTS**	**PC**	**MTS**	**HPC**	**HPTS**	**OxTS**	**OC**	**MX^{-}**
1	-	-	57	i438	59	i29	30	i19	i532	125	208
2			64	146	88	86	106	111	29	127	
3			77	167	91	103	122	139	107	242	
4			639	171	531	549	553	553	252	539	
5			995	916	805	785	809	827	679	749	
6			999	919	808	807	817	854	783	757	
7			1334	983	1193	1222	1235	1227	1107	1210	
8			1470	1403	1440	1443	1443	1440	1429	1467	
9			1473	1406	1441	1457	1460	1467	1472	1472	
10			3111	3154	3092	3049	3035	3008	3124	3011	
11			3225	3342	3205	3159	3151	3137	3237	3069	
12			3228	3345	3206	3178	3172	3161	3259	3076	

Table S10 Continues

**Au^- + CH_3Br**	**XC**	**XTS**	**RC**	**invTS**	**PC**	**MTS**	**HPC**	**HPTS**	**OxTS**	**OC**	**MX^-**
1	54	i38	55	i387	40	-	-	i17	i450	110	140
2	73	13	81	105	59			88	60	112	
3	76	83	92	146	80			107	75	160	
4	578	643	501	153	532			553	247	530	
5	921	968	938	887	790			826	659	743	
6	924	972	942	893	799			843	749	749	
7	1289	1331	1275	965	1188			1226	1091	1203	
8	1480	1485	1462	1410	1439			1442	1435	1468	
9	1481	1489	1462	1412	1443			1464	1464	1470	
10	3079	3098	3119	3150	3087			3020	3128	3018	
11	3178	3199	3245	3333	3200			3142	3243	3081	
12	3181	3203	3249	3340	3201			3164	3262	3083	
**Au^- + CH_3I**	**XC**	**XTS**	**RC**	**invTS**	**PC**	**MTS**	**HPC**	**HPTS**	**OxTS**	**OC**	**MX^-**
1	52	i66	49	-334	36	-	-	i29	i448	97	108
2	84	25	84	88	40			54	57	101	
3	94	107	89	122	50			58	61	122	
4	420	579	398	125	535			554	233	521	
5	780	893	871	845	782			824	623	731	
6	788	897	873	855	788			836	682	737	
7	1173	1272	1194	951	1184			1225	1044	1192	
8	1466	1476	1450	1415	1441			1443	1431	1466	
9	1468	1483	1451	1421	1445			1461	1456	1468	
10	3070	3096	3132	3143	3082			3034	3123	3015	
11	3178	3205	3270	3318	3195			3150	3244	3081	
12	3183	3205	3273	3333	3198			3166	3259	3081	

Cartesian coordinates of optimized structures

5
CH3F

C	0.000000	0.000000	-0.631567
H	0.000000	1.031016	-0.982185
H	-0.892886	-0.515508	-0.982185
H	0.892886	-0.515508	-0.982185
F	0.000000	0.000000	0.748440

5
CH3Cl

C	0.000000	0.000000	-1.126171
H	0.000000	1.029237	-1.467420
H	-0.891345	-0.514619	-1.467420
H	0.891345	-0.514619	-1.467420
Cl	0.000000	0.000000	0.656429

5
CH3Br

C	0.000000	0.000000	-1.521482
H	0.000000	1.031751	-1.851398
H	-0.893522	-0.515875	-1.851398
H	0.893522	-0.515875	-1.851398
Br	0.000000	0.000000	0.419517

5
CH3I

C	0.000000	0.000000	-1.813235
H	0.000000	1.032908	-2.137696
H	-0.894525	-0.516454	-2.137696
H	0.894525	-0.516454	-2.137696
I	0.000000	0.000000	0.326274

5
CH3Cu

C	0.000000	0.000000	-1.466108
H	0.000000	1.028954	-1.820385
H	-0.891100	-0.514477	-1.820385
H	0.891100	-0.514477	-1.820385
Cu	0.000000	0.000000	0.491648

5
CH3Ag

C	0.000000	0.000000	-1.774085
H	0.000000	1.030696	-2.117231
H	-0.892609	-0.515348	-2.117231
H	0.892609	-0.515348	-2.117231
Ag	0.000000	0.000000	0.361621

5
CH3Au

C	0.000000	0.000000	-1.791244
H	0.000000	1.033358	-2.128046
H	-0.894915	-0.516679	-2.128046
H	0.894915	-0.516679	-2.128046
Au	0.000000	0.000000	0.216856

4
CH3-radical

C	0.000000	0.000000	0.000000
H	0.000000	1.076701	0.000000
H	-0.932450	-0.538350	0.000000
H	0.932450	-0.538350	0.000000

2
CuF-ion

Cu	0.000000	0.000000	0.444942
F	0.000000	0.000000	-1.433703

2
CuCl-ion

Cu	0.000000	0.000000	0.841632
Cl	0.000000	0.000000	-1.435725

2
CuBr-ion

Cu	0.000000	0.000000	-1.325623
Br	0.000000	0.000000	1.098373

2
CuI-ion

I	0.000000	0.000000	0.927456
Cu	0.000000	0.000000	-1.695005

2
AgF-ion

Ag	0.000000	0.000000	0.343859
F	0.000000	0.000000	-1.795706

2
AgCl-ion

Ag	0.000000	0.000000	0.670782
Cl	0.000000	0.000000	-1.854516

2
AgBr-ion

Ag	0.000000	0.000000	1.139991
Br	0.000000	0.000000	-1.530846

2
AgI-ion

Ag	0.000000	0.000000	-1.510749
I	0.000000	0.000000	1.339721

2
AuF-ion

Au	0.000000	0.000000	0.217520
F	0.000000	0.000000	-1.909344

2
AuCl-ion

Au	0.000000	0.000000	0.443080
Cl	0.000000	0.000000	-2.059019

2
AuBr-ion

Au	0.000000	0.000000	0.804820
Br	0.000000	0.000000	-1.816593

2
AuI-ion

I	0.000000	0.000000	-1.665608
Au	0.000000	0.000000	1.117433

2
HCu

Cu	0.00000000	0.00000000	0.05077400
H	0.00000000	0.00000000	-1.47243700

2
HAg

Ag	0.00000000	0.00000000	0.03441000
H	0.00000000	0.00000000	-1.61726400

2
HAu

Au	0.00000000	0.00000000	0.01912100
H	0.00000000	0.00000000	-1.51053500

4
CH2F-ion

C	0.07554900	0.75607900	0.00000000
H	-0.56662100	0.99116400	0.87360700
H	-0.56662100	0.99116400	-0.87360700
F	0.07554900	-0.72431100	0.00000000

4
CH2Cl-ion

C	0.05249300	1.29184200	0.00000000
H	-0.60366900	1.47068300	0.86774900
H	-0.60366900	1.47068300	-0.86774900
Cl	0.05249300	-0.62896600	0.00000000

4
CH2Br-ion

C	0.03107800	1.69616900	0.00000000
H	-0.63709500	1.84316800	0.86566700
H	-0.63709500	1.84316800	-0.86566700
Br	0.03107800	-0.39609600	0.00000000

4
CH2I-ion

C	0.02206100	1.97861900	0.00000000
H	-0.65081300	2.11029600	0.86526000
H	-0.65081300	2.11029600	-0.86526000
I	0.02206100	-0.30362800	0.00000000

6
CuF-RC

C	0.007255	-2.035916	0.000000
H	1.038875	-1.691263	0.000000
H	-0.502760	-1.673468	0.889749
H	-0.502760	-1.673468	-0.889749
F	-0.008543	-3.435114	0.000000
Cu	0.000000	1.661025	0.000000

6
CuF-invTS

C	-1.012328	0.000130	0.000433
H	-1.054608	-0.831785	-0.677417
H	-1.055933	-0.170992	1.059741
H	-1.054681	1.003136	-0.381063
Cu	1.246774	-0.000019	-0.000050
F	-2.990807	-0.000064	-0.000266

6
CuF-PC

Cu	-0.02489000	0.00428900	0.01530200
C	0.01139000	-0.00090900	1.91893300
F	0.06003200	-0.00820000	4.48019700
H	-0.50477700	-0.89511400	2.25879900
H	-0.49450800	0.89736700	2.26347600
H	1.05242400	-0.00769200	2.23157400

6
CuF-MTS

Cu	0.03544800	0.00317500	0.01337900
C	-0.01364700	-0.00073900	1.91458200
F	0.30390600	-0.00847300	4.47986300
H	-0.54485200	-0.89781600	2.22530600
H	-0.54043600	0.89767400	2.22900600
H	1.00410700	-0.00407800	2.29626900

6
CuF-HPC

C	-0.90474400	0.60079200	0.00000000
H	-0.10129900	1.39560900	0.00000000
H	-1.53051800	0.61818500	0.89596700
H	-1.53051800	0.61818500	-0.89596700
F	0.95453300	2.72309000	0.00000000
Cu	0.00000000	-1.06015700	0.00000000

6
CuF-HPTS

Cu	0.960836	-0.254589	0.000001
C	-0.336069	1.213060	-0.000003
H	-1.247882	0.566763	-0.000073
H	-0.203644	1.812213	0.899472
H	-0.203490	1.812327	-0.899380
F	-2.688089	-0.454063	-0.000002

6
CuF-OxTS

C	-1.732557	-0.677822	0.001390
H	-1.365344	-1.395175	-0.718748
H	-2.603103	-0.112731	-0.287967
H	-1.659059	-0.972819	1.037222
Cu	0.829081	-0.100696	-0.000607
F	-0.891165	1.051984	-0.002359

6
CuF-OC

C	-1.927674	0.000263	-0.000321
H	-2.337851	0.405152	-0.933359
H	-2.340035	0.606126	0.815551
H	-2.340193	-1.009343	0.115095
F	1.903057	0.000401	-0.000494
Cu	0.050228	-0.000246	0.000313

6
CuCl-RC

C	0.000000	1.315581	0.000000
H	-1.029662	0.978751	0.000000
H	0.513110	0.972505	0.890430
H	0.513110	0.972505	-0.890430
Cu	-0.003384	-2.209698	0.000000
Cl	0.005975	3.133177	0.000000

6
CuCl-invTS

C	-0.660349	-0.000303	-0.001782
H	-0.528336	-0.951076	0.483832
H	-0.528574	0.896433	0.577485
H	-0.529071	0.053851	-1.068122
Cu	1.867202	0.000044	0.000283
Cl	-2.858869	0.000078	0.000547

6
CuCl-PC

Cu	-0.00031400	-0.00017500	0.04166700
C	0.00012400	0.00005900	1.92906000
Cl	0.00030400	-0.00019900	5.27376800
H	-0.73006000	-0.73017200	2.27546300
H	-0.26708400	0.99768200	2.27510400
H	0.99779300	-0.26719500	2.27493700

6
CuCl-MTS

Cu	-1.94187900	-0.14337200	0.00000000
C	-0.15597900	0.65435700	0.00000100
H	-0.06039500	1.26577300	-0.89428600
H	-0.06036000	1.26575400	0.89429700
H	0.59555700	-0.13565400	-0.00001400
Cl	3.33973900	-0.12730700	0.00000000

6
CuCl-OxTS

C	0.80660500	1.51660300	0.00000300
H	0.26980100	1.73255900	-0.91001300
H	1.84584800	1.80048500	-0.00022200
H	0.27019800	1.73242200	0.91028500
Cu	-1.27831900	-0.15481200	-0.00000100
Cl	1.75563300	-0.58091400	-0.00000200

6
CuCl-OC

C	-2.331774	0.000571	0.000515
H	-2.740333	0.618755	-0.806728
H	-2.739212	0.391450	0.939660
H	-2.741011	-1.007525	-0.130348
Cu	-0.341721	-0.000398	-0.000369
Cl	1.889477	0.000320	0.000295

6
CuBr-XC

C	-0.015905	3.049129	0.000000
H	1.012753	3.392698	0.000000
H	-0.534856	3.379150	0.893256
H	-0.534856	3.379150	-0.893256
Cu	0.005255	-2.260075	0.000000
Br	0.000000	1.059897	0.000000

6
CuBr-XTS

Cu	3.80236400	0.08028000	-0.00000500
Br	-2.21059400	-0.35699500	0.00001400
C	-3.57912200	1.05009900	-0.00007200
H	-3.06703900	1.99850500	-0.11339000
H	-4.10692200	1.00206100	0.94541900
H	-4.24906000	0.86556500	-0.83196100

6
CuBr-RC

C	0.00000000	0.38162300	0.00000000
H	-1.01395900	0.00336500	0.00000000
H	0.53512000	0.09445000	0.89580500
H	0.53512000	0.09445000	-0.89580500
Cu	0.14012100	-2.95848400	0.00000000
Br	-0.11770900	2.38040100	0.00000000

6
CuBr-invTS

C	0.036120	0.001312	0.003369
H	0.214756	-0.734395	-0.763047
H	0.213767	-0.295655	1.023734
H	0.214966	1.033210	-0.249454
Cu	2.664046	-0.000184	-0.000509
Br	-2.231930	-0.000163	-0.000477

6
CuBr-PC

C	0.99068500	-0.05118800	0.00126500
H	0.64540300	-0.31191800	1.00122100
H	0.64721800	-0.78845000	-0.72372100
H	0.64171400	0.94360900	-0.27387600
Br	-2.54756700	-0.05582000	0.00315100
Cu	2.87742700	-0.04539100	0.00157400

6
CuBr-HPTS

Cu	2.339612	-0.350297	0.000000
C	1.369195	1.350807	0.000000
H	0.351962	0.951819	-0.000135
H	1.590439	1.920014	0.899374
H	1.590622	1.920143	-0.899246
Br	-2.274198	-0.078234	0.000000

6
CuBr-OxTS

C	-0.09073600	1.74677200	-0.00001500
H	0.47383700	1.86929100	0.91028800
H	-1.06677800	2.20216800	-0.00008500
H	0.47395900	1.86928000	-0.91024000
Br	-1.47293700	-0.27141100	-0.00006500
Cu	1.80055800	-0.23869000	0.00008200

6
CuBr-OC

C	-2.867557	-0.000059	0.000642
H	-3.273480	0.844190	0.568650
H	-3.274598	-0.913781	0.447264
H	-3.274764	0.070369	-1.013893
Cu	-0.874692	-0.000018	-0.000373
Br	1.496979	0.000003	0.000141

6
CuI-XC

C	3.016475	-0.004336	0.000889
H	3.327249	0.352952	-0.975488
H	3.321921	-1.030330	0.178943
H	3.327232	0.661186	0.799660
I	0.666302	0.001450	-0.000291
Cu	-2.185837	-0.001194	0.000240

6
CuI-XTS

Cu	3.313272	-0.119630	0.000016
I	-1.376691	0.332661	-0.000037
C	-2.512233	-1.479760	0.000284
H	-1.820089	-2.283326	0.222117
H	-2.948364	-1.601352	-0.983495
H	-3.278398	-1.398521	0.761177

6
CuI-RC

C	0.381833	-0.003565	0.002107
H	0.651068	0.835830	0.627419
H	0.652578	0.117733	-1.037023
H	0.649831	-0.965006	0.416858
I	-1.913121	0.000343	-0.000212
Cu	3.350033	0.000505	-0.000298

6
CuI-invTS

C	0.413475	-0.011889	-0.000219
H	0.669923	-0.559866	0.894946
H	0.676343	1.035695	0.025280
H	0.669755	-0.515525	-0.921096
I	-1.902790	0.001154	0.000023
Cu	3.322448	0.001720	0.000033

6
CuI-PC

C	1.672510	0.385736	-0.001844
H	1.109816	-0.419844	0.462854
H	1.345249	0.511827	-1.030589
H	1.538391	1.309313	0.555678
Cu	3.573433	-0.085451	0.000522
I	-2.219964	-0.023352	0.000151

6
CuI-HPTS

Cu	2.955621	-0.359931	0.000000
C	2.011076	1.354709	-0.000002
I	-1.948029	-0.047616	0.000000
H	2.241673	1.921403	0.898567
H	2.241852	1.921548	-0.898432
H	0.982560	0.990407	-0.000118

6
CuI-OxTS

C	0.42183900	1.84865400	-0.00000500
H	0.99492200	1.91112800	0.91072900
H	-0.50790300	2.39016600	0.00087100
H	0.99348700	1.91145100	-0.91162900
I	-1.34549000	-0.16928400	0.00000000
Cu	2.32066900	-0.28733200	0.00000100

6
CuI-OC

C	3.273739	-0.000022	0.000278
H	3.678611	-0.743510	0.694983
H	3.678486	0.973422	0.296865
H	3.679098	-0.229877	-0.990756
I	-1.278024	-0.000003	0.000040
Cu	1.277816	0.000009	-0.000169

6
AgF-RC

C	-0.006737	-2.530419	0.000000
H	1.016609	-2.162227	0.000000
H	-0.524600	-2.179601	0.889936
H	-0.524600	-2.179601	-0.889936
Ag	0.000000	1.214187	0.000000
F	0.008113	-3.929203	0.000000

6
AgF-invTS

C	1.400345	0.000039	0.000724
H	1.515627	0.926206	-0.530068
H	1.516001	-0.923936	-0.533819
H	1.516639	-0.002119	1.068189
Ag	-0.945030	-0.000005	-0.000080
F	3.496231	-0.000014	-0.000542

6
AgF-PC

C	0.013149	-1.194468	0.000000
H	-1.019323	-1.514058	0.000000
H	0.532886	-1.501666	0.896639
H	0.532886	-1.501666	-0.896639
Ag	0.000000	0.967581	0.000000
F	-0.013927	-3.754678	0.000000

6
AgF-MTS

C	-1.182234	0.162186	-0.000009
H	-1.601905	-0.834190	0.000288
H	-1.446501	0.704372	-0.898203
H	-1.446434	0.704912	0.897875
Ag	0.968245	-0.017886	0.000001
F	-3.768807	-0.078616	0.000005

6
AgF-HPC

C	0.91077100	-1.07809400	0.00000000
H	0.07231700	-1.83162100	0.00000000
H	1.53013700	-1.09685500	0.89670600
H	1.53013700	-1.09685500	-0.89670600
F	-0.95524600	-3.15878600	0.00000000
Ag	0.00000000	0.82814800	0.00000000

6
AgF-HPTS

C	-1.086228	0.623031	-0.000002
H	-1.815860	-0.202756	-0.000220
H	-1.174624	1.231444	-0.896148
H	-1.174773	1.230963	0.896456
Ag	0.935370	-0.071610	-0.000001
F	-3.697751	-0.292462	-0.000004

6
AgF-OxTS

C	-1.87238100	-0.87715500	0.00199400
H	-1.27524400	-1.67197600	-0.42118700
H	-2.71705900	-0.57658000	-0.59474900
H	-2.02930600	-0.93929700	1.06646800
Ag	0.66579500	-0.02179100	-0.00061200
F	-1.55960800	1.05277300	-0.00375100

6
AgF-OC

C	2.105755	-0.000245	0.000167
H	2.505232	-0.025681	-1.017861
H	2.505336	-0.869290	0.531078
H	2.504820	0.894454	0.486914
Ag	-0.025985	0.000092	-0.000048
F	-2.103180	-0.000262	0.000124

6
AgCl-RC

C	-0.015425	-1.917606	0.000000
H	1.007903	-1.562299	0.000000
H	-0.534185	-1.583494	0.890556
H	-0.534185	-1.583494	-0.890556
Ag	0.000000	1.695382	0.000000
Cl	0.009001	-3.732237	0.000000

6
AgCl-invTS

C	1.114232	0.000002	0.003407
H	1.061052	0.026706	1.075868
H	1.060533	0.915606	-0.555575
H	1.060291	-0.942263	-0.509365
Ag	-1.448946	0.000000	-0.000316
Cl	3.425483	-0.000005	-0.000972

6

AgCl-PC

C	0.000000	0.596011	0.000000
H	1.021327	0.958383	0.000000
H	-0.524207	0.918135	0.892015
H	-0.524207	0.918135	-0.892015
Cl	-0.153470	3.891862	0.000000
Ag	0.056087	-1.543242	0.000000

6

AgCl-HPTS

Ag	-1.212017	-0.215742	0.000000
C	0.054941	1.504492	-0.000006
H	-0.120082	2.085791	-0.900115
H	-0.119388	2.085173	0.900637
H	1.016159	0.986112	-0.000483
Cl	3.285793	-0.237892	-0.000001

6

AgCl-OxTS

C	1.443300	1.586749	0.000001
H	0.959884	1.893012	-0.915058
H	2.519017	1.607801	-0.000076
H	0.960016	1.892866	0.915176
Ag	-0.952802	-0.048123	0.000000
Cl	1.863705	-0.744258	-0.000002

6

AgCl-OC

C	2.439618	-0.001380	0.000410
H	2.833710	-0.445334	0.918368
H	2.837516	1.014013	-0.074811
H	2.833917	-0.576063	-0.841664
Ag	0.289150	0.000619	-0.000176
Cl	-2.160760	-0.000789	0.000232

6

AgBr-XC

C	-0.014828	3.579731	0.000000
H	1.013282	3.924781	0.000000
H	-0.533151	3.912164	0.892654
H	-0.533151	3.912164	-0.892654
Ag	0.003021	-1.901580	0.000000
Br	0.000000	1.604194	0.000000

6

AgBr-XTS

C	3.33283200	1.09230400	0.00000000
H	2.74033000	2.00078900	0.00007900
H	3.94719100	1.03638300	-0.89185800
H	3.94730500	1.03630200	0.89177600
Ag	-2.22357600	0.06418600	0.00000000
Br	2.11074900	-0.38982900	0.00000000

6

AgBr-RC

C	0.000000	1.034479	0.000000
H	-1.023329	0.682266	0.000000
H	0.526882	0.730797	0.895348
H	0.526882	0.730797	-0.895348
Ag	0.046126	-2.432091	0.000000
Br	-0.062810	3.027358	0.000000

6

AgBr-invTS

C	0.445113	-0.000074	-0.002472
H	0.346628	0.922263	-0.546238
H	0.346500	-0.932367	-0.528977
H	0.347016	0.009899	1.068114
Ag	-2.188018	0.000007	0.000227
Br	2.832172	0.000009	0.000322

6

AgBr-PC

C	0.00005400	0.24322000	0.08450700
H	-1.06207400	0.02626900	0.08553100
H	0.46816700	-0.10848700	0.99696900
H	0.47992900	-0.18919700	-0.78577900
Br	-0.32349700	-3.21468200	-0.00578000
Ag	0.24350300	2.36740500	-0.00744700

6

AgBr-HPTS

C	0.38877900	0.73269500	0.00000000
H	-0.32884800	-0.08382700	-0.00040900
H	0.31023900	1.33642500	-0.89893100
H	0.30997600	1.33576900	0.89934800
Ag	2.33210500	-0.09589000	0.00000000
Br	-3.20665600	-0.07079100	0.00000000

6

AgBr-OxTS

C	0.729539	1.950333	0.000000
H	0.194991	2.156923	-0.914537
H	1.776124	2.201994	0.000078
H	0.194849	2.156953	0.914448
Ag	-1.382644	-0.119406	0.000000
Br	1.669745	-0.360165	0.000000

6

AgBr-OC

C	-2.920711	-0.001529	-0.000921
H	-3.312685	-0.450594	-0.916990
H	-3.316959	1.014643	0.068327
H	-3.314511	-0.571563	0.844248
Ag	-0.764899	0.000649	0.000388
Br	1.811963	-0.000395	-0.000237

6
AgI-XC

C	-0.021692	3.455154	0.000000
H	1.009469	3.791844	0.000000
H	-0.542832	3.774766	0.895979
H	-0.542832	3.774766	-0.895979
Ag	0.004390	-2.050416	0.000000
I	0.000000	1.213155	0.000000

6
AgI-XTS

C	3.095747	1.480665	-0.000263
H	2.404792	2.284188	-0.225646
H	3.864164	1.397996	-0.758742
H	3.529129	1.603951	0.984525
Ag	-2.815105	0.072476	-0.000009
I	1.961083	-0.331632	0.000035

6
AgI-RC

C	0.000000	0.314173	0.000000
H	-1.016261	-0.055783	0.000000
H	0.540758	0.058296	0.901127
H	0.540758	0.058296	-0.901127
I	-0.163647	2.543554	0.000000
Ag	0.183150	-2.909664	0.000000

6
AgI-invTS

C	-0.025801	0.012174	-0.000261
H	-0.181650	0.569475	0.908121
H	-0.182703	-1.052718	0.028138
H	-0.181650	0.520252	-0.937063
I	2.460300	-0.001071	0.000023
Ag	-2.759470	-0.001134	0.000024

6
AgI-PC

C	-0.937165	0.303669	0.001863
H	-0.627630	0.476963	1.027067
H	-0.734213	1.169786	-0.619313
H	-0.451356	-0.579776	-0.397577
Ag	-3.046658	-0.039075	-0.000295
I	2.842059	-0.019858	-0.000141

6
AgI-HPTS

C	1.377092	1.572017	0.000002
H	0.375885	1.143417	-0.001213
H	1.592039	2.139801	-0.899782
H	1.590153	2.138733	0.900884
Ag	2.507407	-0.242950	0.000003
I	-2.446580	-0.064819	-0.000001

6
AgI-OxTS

C	0.160061	2.043351	0.000001
H	-0.398190	2.174416	-0.914170
H	1.152195	2.459902	-0.000176
H	-0.397884	2.174420	0.914360
Ag	-1.848842	-0.170314	0.000000
I	1.614700	-0.208756	0.000000

6
AgI-OC

C	-3.308709	0.000152	0.000483
H	-3.700852	0.277861	-0.980827
H	-3.701214	0.711198	0.731331
H	-3.701420	-0.988359	0.250504
Ag	-1.146798	-0.000061	-0.000155
I	1.601042	0.000024	0.000063

6
AuF-RC

C	-0.012845	-2.704475	0.000000
H	1.005791	-2.325576	0.000000
H	-0.533595	-2.358802	0.889472
H	-0.533595	-2.358802	-0.889472
F	0.015386	-4.107626	0.000000
Au	0.000000	0.762515	0.000000

6
AuF-invTS

C	1.664291	0.000094	-0.000001
H	1.737137	1.071912	-0.012378
H	1.736937	-0.525128	0.934389
H	1.736936	-0.546568	-0.922013
F	3.620801	-0.000053	0.000001
Au	-0.604860	-0.000004	0.000000

6
AuF-PC

C	-0.011728	-1.417803	0.000000
H	-1.047433	-1.733401	0.000000
H	0.503136	-1.745286	0.894170
H	0.503136	-1.745286	-0.894170
F	0.012392	-3.917843	0.000000
Au	0.000000	0.620144	0.000000

6
AuF-MTS

C	0.273295	-1.390548	0.000000
H	-0.681374	-1.908293	0.000000
H	0.834371	-1.631426	0.897163
H	0.834371	-1.631426	-0.897163
F	-0.291904	-3.933070	0.000000
Au	-0.000000	0.619140	0.000000

6
AuF-HPC

C	0.872042	-1.279710	0.000000
H	0.055835	-2.070508	0.000000
H	1.497025	-1.303618	0.894139
H	1.497025	-1.303618	-0.894139
Au	0.000000	0.537943	0.000000
F	-0.920238	-3.349056	0.000000

6
AuF-HPTS

C	-0.836991	1.470024	0.000000
H	-0.645187	2.058018	0.896746
H	-0.645187	2.058018	-0.896746
H	-1.794936	0.909964	0.000000
F	-2.748469	-0.611974	0.000000
Au	0.415740	-0.105549	0.000000

6
AuF-OxTS

C	1.786340	0.896101	-0.000017
H	1.086147	1.735377	0.001687
H	2.373083	0.913840	-0.909500
H	2.376446	0.913202	0.907325
Au	-0.438669	-0.011111	0.000004
F	2.011235	-0.895699	0.000033

6
AuF-OC

C	-2.008737	-0.000083	-0.000331
H	-2.415833	-0.715967	0.721647
H	-2.414732	-0.268265	-0.981552
H	-2.416730	0.983019	0.257735
Au	0.007455	0.000038	0.000101
F	2.078972	-0.000148	-0.000426

6
AuCl-RC

C	-0.018516	-2.255103	0.000000
H	1.002789	-1.896516	0.000000
H	-0.537809	-1.922812	0.889968
H	-0.537809	-1.922812	-0.889968
Au	0.000000	1.120736	0.000000
Cl	0.010819	-4.074436	0.000000

6
AuCl-invTS

C	-1.548671	-0.000127	-0.001456
H	-1.458559	-0.945973	0.499149
H	-1.458994	0.906927	0.566405
H	-1.458934	0.038759	-1.070917
Cl	-3.745191	0.000028	0.000440
Au	0.978946	0.000007	0.000084

6
AuCl-PC

C	0.000000	-1.002197	0.000000
H	-1.028507	-1.347406	0.000000
H	0.519594	-1.332922	0.892975
H	0.519594	-1.332922	-0.892975
Au	-0.020139	1.019655	0.000000
Cl	0.092960	-4.148605	0.000000

6
AuCl-MTS

C	0.761254	-0.870007	0.000000
H	-0.052851	-1.595831	0.000000
H	1.367951	-0.968368	0.896890
H	1.367951	-0.968368	-0.896890
Au	0.000000	0.994829	0.000000
Cl	-0.426505	-4.108171	0.000000

6
AuCl-HPC

C	0.921470	-0.818427	0.000000
H	0.165382	-1.607942	0.000000
H	1.535614	-0.859481	0.896858
H	1.535614	-0.859481	-0.896858
Au	0.000000	0.970408	0.000000
Cl	-0.515614	-4.024986	0.000000

6
AuCl-HPTS

C	-0.372575	1.500597	0.000000
H	-0.145236	2.073358	0.896806
H	-0.145235	2.073358	-0.896805
H	-1.392763	1.103584	0.000000
Cl	-3.504059	-0.231443	0.000000
Au	0.803642	-0.130625	0.000000

6
AuCl-OxTS

C	1.374595	1.517907	0.001247
H	0.714383	1.887349	-0.769842
H	2.410933	1.745442	-0.190236
H	1.056550	1.736075	1.009602
Cl	2.417538	-0.564808	-0.002033
Au	-0.677564	-0.061703	-0.000284

6
AuCl-OC

C	-2.243497	0.000660	0.000617
H	-2.644565	0.694592	-0.743803
H	-2.642873	0.299406	0.974229
H	-2.644422	-0.991414	-0.226891
Au	-0.207667	-0.000156	-0.000167
Cl	2.223444	0.000338	0.000352

6

AuBr-XC

C	0.006353	-3.954048	0.000000
H	-1.021586	-4.301081	0.000000
H	0.522697	-4.294050	0.891523
H	0.522697	-4.294050	-0.891523
Au	0.000000	1.343336	0.000000
Br	-0.001769	-1.986002	0.000000

6

AuBr-XTS

Au	-1.677952	0.038161	-0.000010
Br	2.742414	-0.388794	0.000062
C	3.992633	1.090175	-0.000357
H	4.265982	1.301617	1.026959
H	3.488513	1.940831	-0.444395
H	4.863456	0.809597	-0.581778

6

AuBr-RC

C	0.000000	1.554444	0.000000
H	-1.026249	1.213534	0.000000
H	0.522438	1.242899	0.894214
H	0.522438	1.242899	-0.894214
Au	0.016510	-1.736208	0.000000
Br	-0.037799	3.546699	0.000000

6

AuBr-invTS

C	1.022991	0.000184	-0.000755
H	0.896889	-0.910846	-0.556626
H	0.896792	0.937555	-0.510952
H	0.897671	-0.026218	1.066216
Br	3.312790	-0.000023	0.000083
Au	-1.579455	-0.000010	0.000038

6

AuBr-PC

-1 1

C	0.000000	0.345672	0.000000
H	1.021780	0.711543	0.000000
H	-0.526516	0.665883	0.893271
H	-0.526516	0.665883	-0.893271
Au	0.060753	-1.674475	0.000000
Br	-0.136235	3.661891	0.000000

6

AuBr-HPTS

C	0.223140	1.494283	0.000000
H	0.448892	2.067114	0.896900
H	0.448893	2.067115	-0.896899
H	-0.800440	1.110466	-0.000001
Br	-3.209875	-0.105167	0.000000
Au	1.403917	-0.133285	0.000000

6

AuBr-OxTS

C	0.801518	1.762696	0.000170
H	0.235596	1.984519	-0.891969
H	1.812793	2.134110	-0.028091
H	0.289292	1.968549	0.927846
Br	2.209494	-0.271888	-0.000153
Au	-1.069356	-0.090472	-0.000044

6

AuBr-OC

C	-2.611478	-0.001254	0.001826
H	-3.010588	0.995925	-0.201459
H	-3.005788	-0.324117	0.968954
H	-3.008691	-0.678125	-0.759038
Au	-0.569504	0.000313	-0.000443
Br	1.990993	-0.000312	0.000444

6

AuI-XC

C	3.783183	0.000634	0.005395
H	4.118112	1.030238	0.082927
H	4.122815	-0.447734	-0.922901
H	4.116535	-0.580005	0.859805
I	1.549175	-0.000204	-0.001717
Au	-1.483074	0.000057	0.000491

6

AuI-XTS

Au	0.000000	1.946558	0.000000
I	-0.293221	-2.337718	0.000000
C	1.626708	-3.274177	0.000000
H	2.145454	-2.942706	0.891106
H	2.145454	-2.942706	-0.891106
H	1.489542	-4.348557	0.000000

6

AuI-RC

C	0.960957	0.003339	0.004951
H	0.661300	0.566885	-0.866661
H	0.666919	0.478542	0.929268
H	0.659935	-1.033373	-0.044989
I	3.178058	-0.000359	-0.000527
Au	-2.230266	-0.000165	-0.000246

6

AuI-invTS

C	0.598950	-0.005180	0.000873
H	0.437303	1.056770	0.057776
H	0.435952	-0.585723	0.891867
H	0.437232	-0.487025	-0.947305
I	3.028842	0.000454	-0.000070
Au	-2.094086	0.000291	-0.000049

6
AuI-PC

C	0.205367	-0.018931	-0.009173
H	-0.128148	0.266433	-1.001951
H	-0.144024	0.691142	0.733659
H	-0.121190	-1.024840	0.235425
I	-3.332174	0.001255	0.000606
Au	2.224891	0.001447	0.000706

6
AuI-HPTS

C	0.748396	1.480086	0.000000
I	-3.016435	-0.064996	0.000000
H	0.966683	2.055270	0.896930
H	0.966696	2.055280	-0.896920
H	-0.273555	1.097300	-0.000009
Au	1.945834	-0.134729	0.000000

6
AuI-OxTS

C	0.376368	1.880657	-0.000004
H	-0.178458	2.040154	-0.911740
H	1.352652	2.334849	0.000548
H	-0.179493	2.040333	0.911066
I	2.081023	-0.172220	0.000002
Au	-1.437305	-0.108501	0.000001

6
AuI-OC

C	2.933251	0.000403	-0.000908
H	3.328380	-0.001467	1.017933
H	3.326173	0.884672	-0.508838
H	3.327811	-0.880767	-0.512795
Au	0.883719	-0.000105	0.000203
I	-1.837654	0.000066	-0.000130
